# Supplementary material for: Single-cell profiling of healthy human kidney reveals features of sex-based transcriptional programs and tissue-specific immunity
Source: Nat Commun. 2022 Dec 10;13:7634. doi: 10.1038/s41467-022-35297-z (PMC9741629; doi:10.1038/s41467-022-35297-z)
Supplement: Supplementary file 1 — Supplementary Information [file 41467_2022_35297_MOESM1_ESM.pdf]

Supplementary Data

Title: Single-cell profiling of healthy human kidney reveals features of sex-based transcriptional programs and tissue-specific immunity

Author List: Caitriona M. McEvoy†[1,2,3], Julia M. Murphy†[1,2,4], Lin Zhang[5,6], Sergi Clotet-Freixas[1,2], Jessica A. Mathews[1,2], James An[1,2,4], Mehran Karimzadeh[6,7], Delaram Pouyabahar[8,9], Shenghui Su[1,2], Olga Zaslaver[8,9], Hannes Röst[8,9], Rangi Arambewela[1,2], Lewis Y. Liu[1,2,4], Sally Zhang[10], Keith A. Lawson[10], Antonio Finelli[10,11], Bo Wang[6,7,12,13], Sonya A. MacParland[1,2,4,13], Gary D. Bader[8,9,12,14,15], Ana Konvalinka[*,1,2,3,11,13], Sarah Q. Crome[*,1,2,4]

Affiliations:
[1]Toronto General Hospital Research Institute, University Health Network; Toronto, ON, Canada.
[2]Ajmera Transplant Centre, University Health Network; Toronto, ON, Canada.
[3]Department of Medicine, Division of Nephrology, University Health Network; Toronto, ON, Canada.
[4]Department of Immunology, University of Toronto; Toronto, ON, Canada.
[5]Department of Statistical Sciences, University of Toronto; Toronto, ON, Canada.
[6]Peter Munk Cardiac Centre, University Health Network; Toronto, ON, Canada.
[7]Vector Institute; Toronto, ON, Canada.
[8]Department of Molecular Genetics, University of Toronto; Toronto, ON, Canada.
[9]The Donnelly Centre, University of Toronto; Toronto, ON, Canada.
[10]Division of Urology, Princess Margaret Cancer Centre, University Health Network; Toronto, ON, Canada
[11]Institute of Medical Science, University of Toronto; Toronto, ON, Canada.
[12]Department of Computer Science, University of Toronto; Toronto, ON, Canada.
[13]Department of Laboratory Medicine and Pathobiology, University of Toronto; Toronto, ON, Canada.
[14]Princess Margaret Cancer Centre, University Health Network; Toronto, ON, Canada
[15]The Lunenfeld-Tanenbaum Research Institute, Mount Sinai Hospital; Toronto, ON, Canada

†These authors contributed equally
[*] These authors jointly supervised this work

Supplementary Tables

(Y, yes; N, no; γ-GTP, gamma-glutamyl transpeptidase).

Catalog #	Lot #	Donor ID	Sex	Age (years)	Diabetes	Alcohol Use	Smoker	Characterization
CC-2553	664995	Male 1	M	52	N	Y	N	γ-GTP positive
CC-2553	682573	Male 2	M	50	N	Y	Y	γ-GTP positive
CC-2553	117405	Male 3	M	57	N	N	N	γ-GTP positive
CC-2553	617045	Female 1	F	55	N	N	Y	γ-GTP positive
CC-2553	635750	Female 2	F	52	N	Y	N	γ-GTP positive
CC-2553	114340	Female 3	F	59	N	N	N	γ-GTP positive

Supplementary Table 1. Primary PT donor characteristics. Characteristics of the donors from which primary proximal tubular epithelial cells were isolated for metabolic studies.Y = yes, N = no, g-GTP positive= gamma-glutamyl transpeptidase

Study ID	Group	Donor Age	Donor Sex	Experiment	samples used in scRNAseq)
HKB10	CD45	49	Female	scRNAseq	CD45_1
HKB11	CD45	37	Female	scRNAseq	CD45_2
HKB13	CD45	55	Female	scRNAseq	CD45_3
HKB14	Total	35	Male	scRNAseq	Total1
HKB15	Total	65	Male	scRNAseq	Total2
HKB16	Total	57	Female	scRNAseq	Total3
HKB17	Total	58	Female	scRNAseq	Total4
HKB18	Total	64	Female	scRNAseq	Total5
HKB19	CD45	43	Male	scRNAseq	CD45_4
HKB21	CD45	34	Male	scRNAseq	CD45_4
HKB23	CD45	38	Male	scRNAseq	CD45_5
HKB24	CD45	35	Female	scRNAseq	CD45_6
HKB26	CD45	43	Female	scRNAseq	CD45_7
HKB27	CD45	42	Male	scRNAseq	CD45_8
HKB28	CD45	41	Male	scRNAseq	CD45_9
HKB31	Total	50	Female	scRNAseq	Total6
HKB32	Total	44	Male	scRNAseq	Total7
HKB33	Total	60	Female	scRNAseq	Total8
HKB34	Total	33	Male	scRNAseq	Total9
HKB38		58	Male	scRNAseq	
HKB38		58	Male	snRNAseq	
RG1655	PBMC	23	Female	Flow Cytometry	
RG1867	PBMC	24	Male	Flow Cytometry	
RG2225	PBMC	56	Female	Flow Cytometry	
RG1879	PBMC	35	Female	Flow Cytometry	
RG2017	PBMC	64	Male	Flow Cytometry	
Leukopak2	PBMC	29	Female	Flow Cytometry	
CBS8120	PBMC	69	Female	Flow Cytometry	
RG2670	PBMC	42	Male	Flow Cytometry	
CBS6920	PBMC	67	Female	Flow Cytometry	
CBS7220	PBMC	70	Female	Flow Cytometry	
Leukopak 4	PBMC	56	Female	Flow Cytometry	
Nephrectomy	Neph	50	Female	Flow Cytometry	
Nephrectomy	Neph	78	Male	Flow Cytometry	
Nephrectomy	Neph	53	Male	Flow Cytometry	
Nephrectomy	Neph	66	Male	Flow Cytometry	
Nephrectomy	Neph	64	Male	Flow Cytometry	
LD 20Ju19	LD	35	Male	Flow Cytometry	
LD 26Ju19	LD	65	Male	Flow Cytometry	
LD 27Ju19	LD	57	Female	Flow Cytometry	
LD 3 Jl19	LD	46	Female	Flow Cytometry	
LD 4Jl19	LD	58	Female	Flow Cytometry	
LD 13D18	LD	58	Male	Flow Cytometry	
LD 17Ap19	LD	32	Female	Flow Cytometry	
LD 26Mr19	LD	55	Male	Flow Cytometry	
LD 20Mr19	LD	51	Male	Flow Cytometry	
LD 31J19	LD	57	Male	Flow Cytometry	

Supplementary Table 2. Human sample donor characteristics. Characteristics of the donors of specimens used for scRNAseq, snRNAseq and flow cytometry

Species	Gene	Gene name	Forward primer (5' → 3')	Reverse primer (5' → 3')
Human	Lysine-specific demethylase 5D	**KDM5D**	GGCTCCCTGCTACGATCACA	CCTCATTGTCAAACGGGTGTGT
Human	Histone demethylase UTY	**UTY**	TGGAGGACCTAATCCAAGTTTATGA	TGCAGAAATTTCCTGAAGAGCA
Human	Eukaryotic translation initiation factor 1A, Y-chromosomal	**EIF1AY**	TGGACGATTGGAAGCATTGTG	AGTCCCGTAGACCAACCAAT
Human	Eukaryotic translation initiation factor 1A, X-chromosomal	**EIF1AX**	CCGCTACCCGGAAAGAAGTCA	ACCCCTGCGTCTGTTTTTACC
Human	ATP-dependent RNA helicase DDX3X	**DDX3X**	TTGCAGTGGAAAATGCGCTC	TATAGCGCCCTTTGCTGGCT
Human	Metallothionein-1F	**MT1F**	AGTCTCTCCTCGGCTTGC	ACATCTGGGAGAAAGGTTGTC
Human	Metallothionein-1G	**MT1G**	CATCTGCAAAGGGGCATCGG	CACTCTATTTGTACTTGGGAGGAG
Human	Metallothionein-1H	**MT1H**	TGGGAACTCCAGTCTCACCT	GTTTTCATCTGACAGCAGGGC
Human	60S ribosomal protein L31	**RPL31**	GCCGTTCTGCCATCAACGAA	TTGAGCCTGGTGTCAATGCG

Supplementary Table 3. qPCR sequences. Primer sequences used for qPCR validation of sex differences in PT cells.

Supplementary Figures

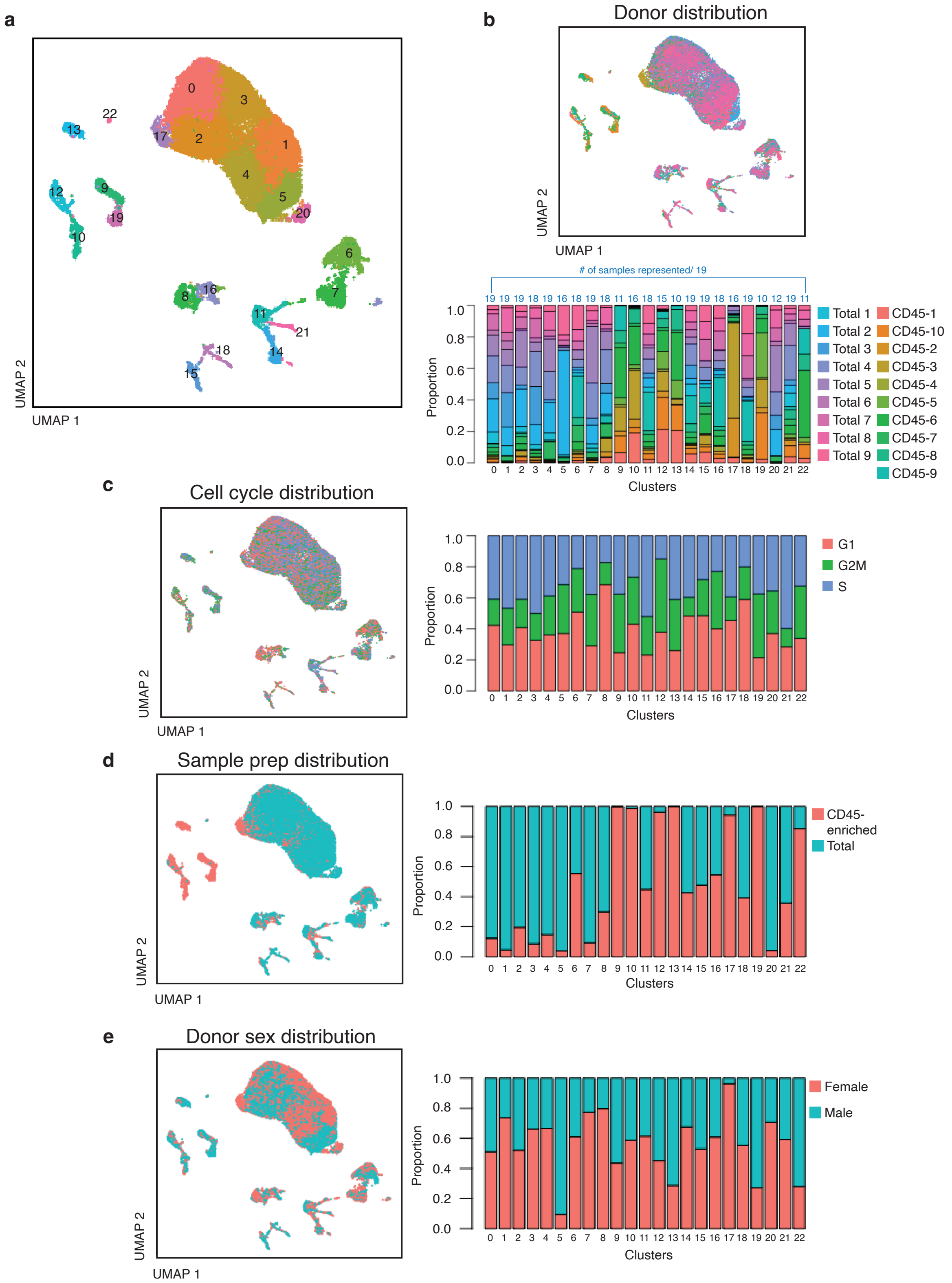

Supplementary Figure 1. Additional proportion plots of total kidney dataset. (**a**) Clustering of total combined dataset of 27677 cells results in 23 clusters (**b**) Individual sample contribution to clustering, demonstrating that clusters are comprised of cells captured from multiple donors and in most cases all 19 samples contribute to each cluster. (**c**) Cell cycle assignment of clusters, with no exceptional variability in cell cycle state across clusters. (**d**) Distribution of sample preparation method (total homogenate versus CD45-positive magnetic bead enrichment) across clusters. **(e**) Distribution of donor sex across clusters. Source data for (b-f) are provided as a source data file.

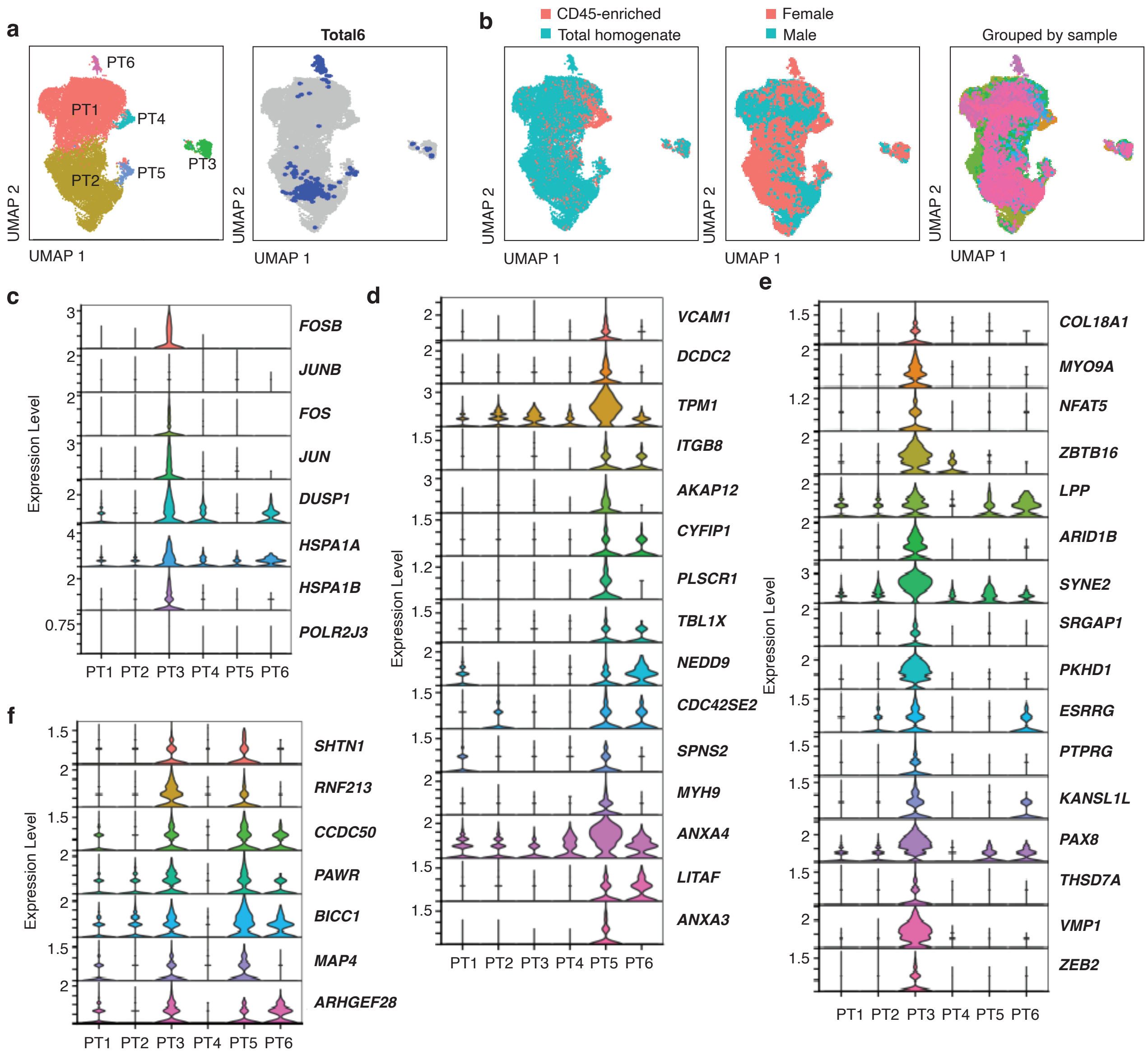

g

CHEA3-Rank	Transcription Factor	Overlapping genes
**PT3**		
1	POU5F1B	38
2	ZNF581	56
3	PA2G4	22
4	ATF3	81
5	ZBED6	12
6	HES4	44
7	EGR1	82
8	FOS	83
9	NFE2L2	67
10	JUN	87

CHEA3-Rank	Transcription Factor	Overlapping genes
**PT5**		
1	ZNF217	20
2	ELF3	12
3	NKX25	3
4	FOSL1	12
5	HOXB2	9
6	NFE2L2	14
7	RFX8	4
8	SNAI2	11
9	PBX3	14
10	ZNF581	7

Supplementary Figure 2. Heterogeneity within Proximal Tubular (PT) dataset. (**a**) Subclustering of PT dataset yielded 6 clusters; PT6 is predominantly composed of cells from one donor: "Total6". (**b**) Distribution of sample preparation method, sex, and donor identity across the PT dataset; PT4 is composed of cells from CD45-enriched samples. (**c**) Stacked violin plots showing enrichment of dissociation stress markers in PT3. (**d**-**f**) Stacked violin plots showing markers of the 'scattered tubular cell' and 'failed PT repair' population enriched in PT5(**d**), PT3(**e**), and both PT3 and PT5(**f**). (**g**) Transcription factor analysis using CHEA3, which illustrates the top 10 transcription factors predicted to regulate PT3, and separately, PT5 genes.

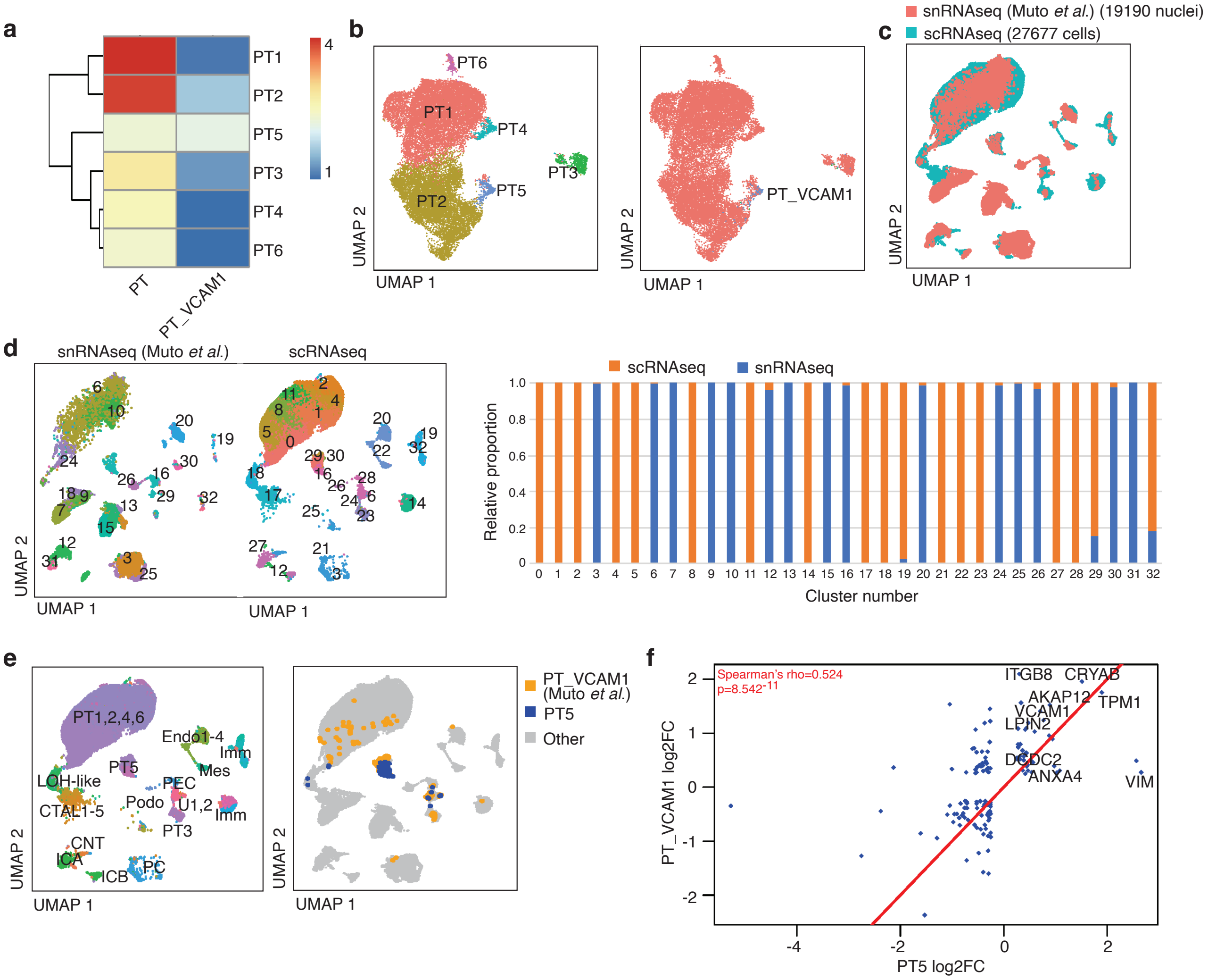

Supplementary Figure 3. Similarity between PT5 cluster with previously published PT_VCAM1 cluster. (**a**) When compared to our scRNAseq data, the 'PT_VCAM1' cluster from Muto *et al*. correlated most highly with our *VCAM1*$^{+}$ PT5 population. **(b)** Unbiased cell-type recognition using the PT cell annotations from the Muto dataset as a reference demonstrates that 'PT_VCAM1' cells identified in Muto *et al*. derive from PT5. (**c**) The snRNAseq data from Muto *et al.* was integrated with our scRNAseq data. (**d**) Despite spatial concordance, clusters remained technology-specific with each cluster being almost exclusively contributed to from one technology/dataset or another. Orange = scRNAseq, blue = snRNAseq. Source data are provided as a source data file. (**e**) Based on annotations in our scRNAseq data, the nuclei from PT_VCAM1 associated mainly with PT5, but also with PT3 and unknown epithelial populations (U1, U2). (**f**) There is a significant positive correlation between the cluster defining genes of PT5 and PT_VCAM1 (Spearman's rho = 0.524, p = 8.542e-11). Source data are provided as a source data file. scRNAseq = single-cell RNA sequencing, snRNAseq = single-nucleus RNA sequencing.

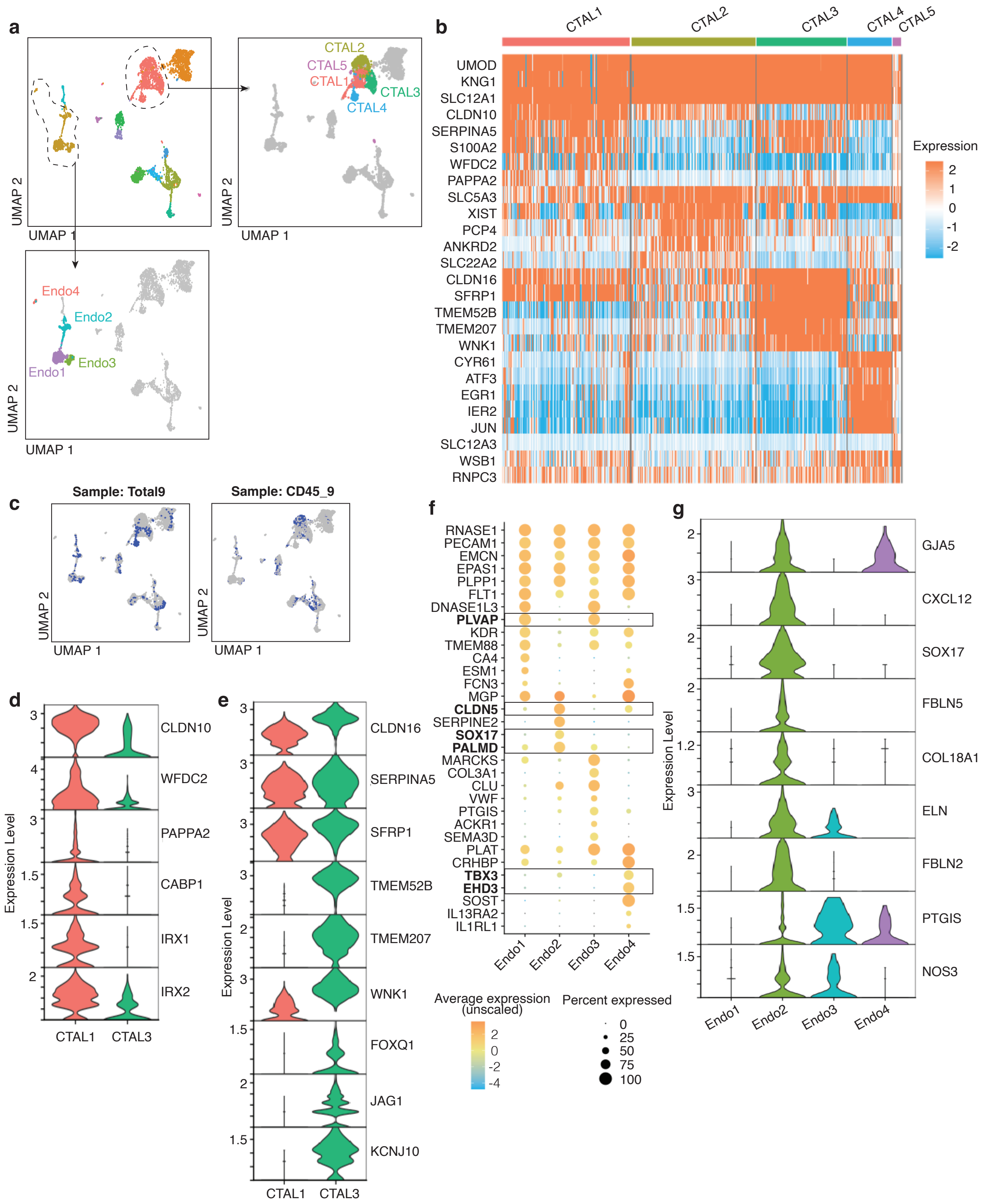

Supplementary Figure 4. Heterogeneity in CTAL and Endothelial cell populations. (**a**) 5 CTAL clusters and 4 endothelial clusters were identified. (**b**) Heatmap depicting expression of the marker genes of CTAL1-5. (**c**) CTAL2 and 4 are each chiefly comprised of cells from one donor (Total9 and CD45_9, respectively). Selected marker genes of *CLDN10*-enriched CTAL1 (**d**) and *CLDN16*- enriched CTAL3 (**e**) populations, respectively. (**f**) Bubble plot showing enrichment for specific endothelial cell markers in all subpopulations; expression of peritubular capillary markers (*PLVAP, TMEM88, DNASE1L3*) in Endo1 and Endo3 respectively; expression of afferent arteriole and vasa recta genes (S*OX17*, *SERPINE2*, *CLDN5*, *CXCL12* and reduced *KDR*) in Endo2; and expression of glomerular microvascular endothelial cell markers in Endo4 (*EDH3*, *SOST* and *TBX3*).(**g**) Increased expression of extracellular matrix genes seen in Endo2 (characterised as afferent arterioles and vasa recta). Of the two peritubular populations described (Endo1 and Endo3), Endo3 has higher expression of vasodilator genes (*PTGIS* and *NOS3*) than Endo1. Endo4 illustrates expression of *GJA5* and *PTGIS*.

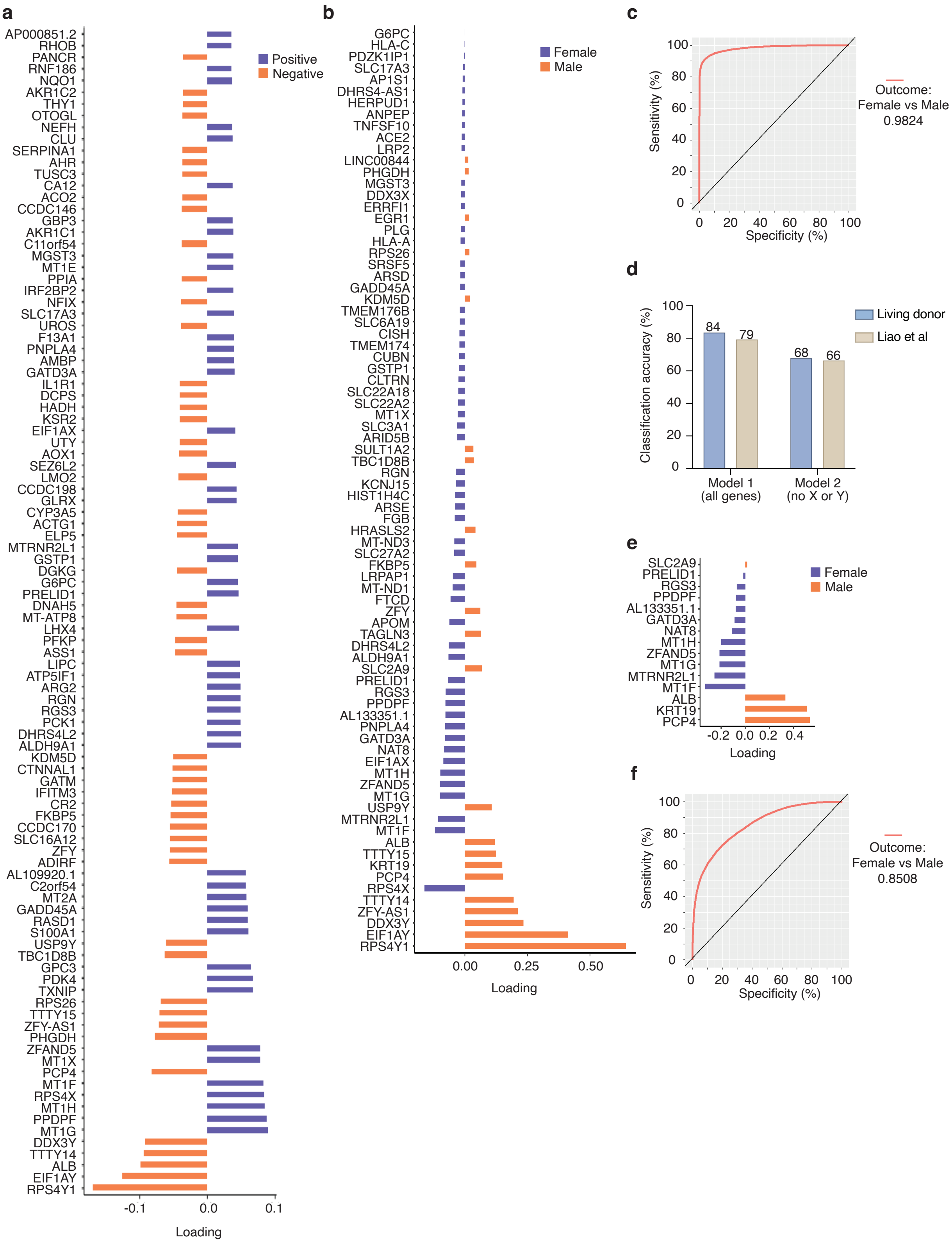

Supplementary Figure 5. Varimax PCA and sparse partial least squares discriminant analysis (sPLS-DA) identifies sex differences in proximal tubular (PT) epithelial cells. (**a**) Top 100 genes (50 from each end of the component) associated with varimax-rotated principal component 12 which revealed sex differences in proximal tubule cells. Orange = male, blue = female. (**b**) Plot of 80 genes that were selected as variables in the sPLS-DA classifier (Model 1) from all detected genes. Orange = male, blue = female. (**c**) Receiver operating characteristic (ROC) curve from Model 1 predict male and female sex with accuracy of 98%. (**d**) Barplot of classification accuracy using Model 1 versus Model 2 to classify PT cells of the living donor data and of a validation dataset from Liao *et al.*[20] Light blue= living donor, tan = Liao et al. (**e**) Plot of 15 genes in Model 2 (using all detected genes except those encoded on X or Y chromosomes as input) where 15 genes were selected as variables in the classifier. Orange = male, blue = female. (**f**) ROC curve from Model 2. Source data for (a, b and e) are provided as a source data file.

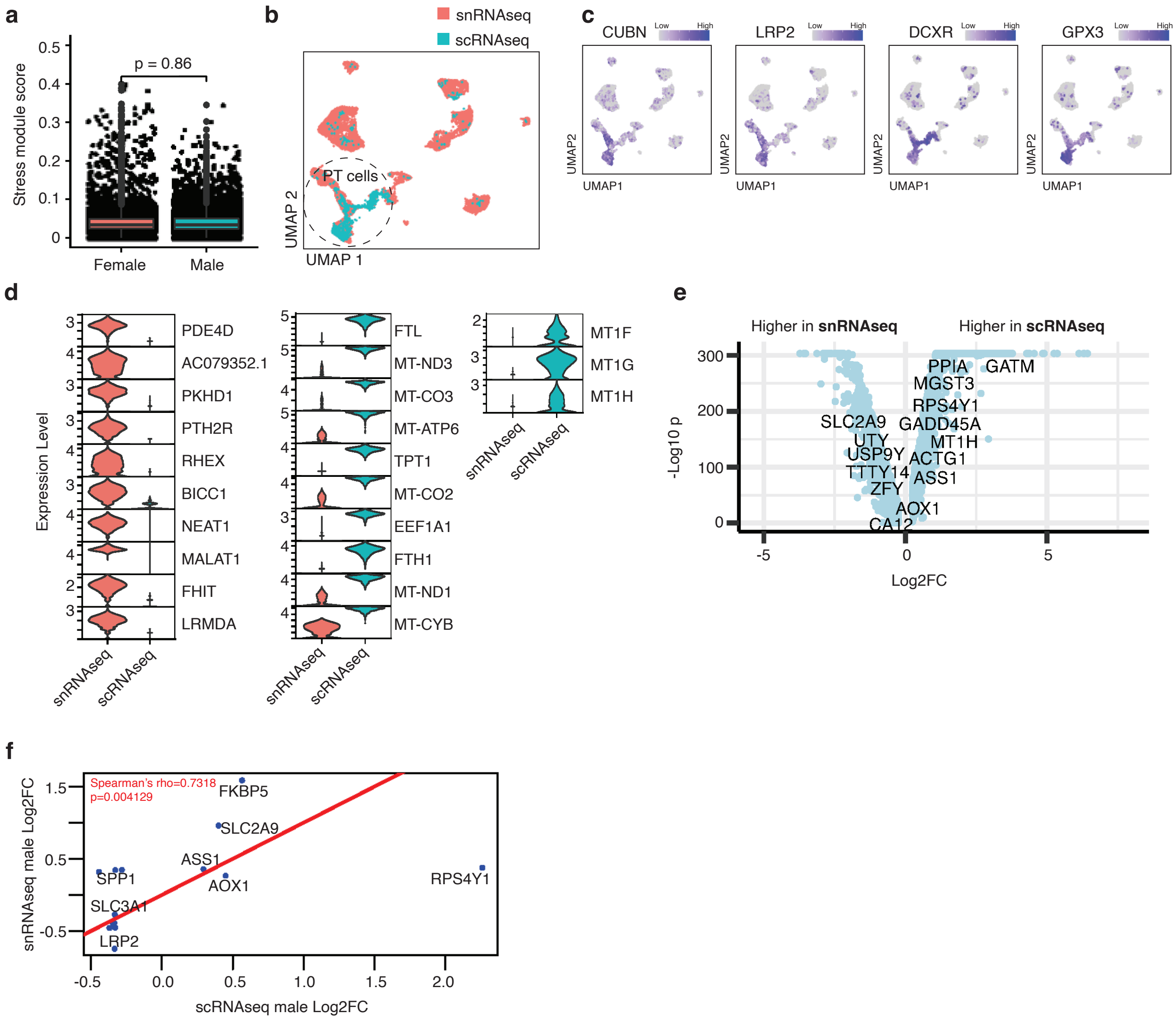

Supplementary Figure 6. Evaluation of dissociation stress and comparison of single nucleus RNA sequencing and single cell RNA sequencing. (**a**) There is no difference in expression of a gene module associated with stress due to warm collagenase dissociation between male and female PT cells (p = 0.86). Differences were assessed by unpaired Wilcoxon test. Pink = female, blue = male. Source data are rpovided as a source data file. (**b**) Data integration from a pilot sequencing experiment in which a single biopsy from a male donor was divided and subjected to scRNAseq and snRNAseq. From the integrated data, PT cell clusters were identified and analyzed. Pink = snRNAseq, blue = scRNAseq. (**c**) Expression of PT cell marker genes used to identify clusters of PT cells in the integrated datasets. (**d**) Comparison of genes from scRNAseq and snRNAseq reveals large descrepancies in gene expression of PT cells between technologies, shown by the top 10 genes with greatest log fold changes in snRNAseq and in scRNAseq. Of note, metallothionein genes which have sex-biased expression, are nearly exclusive to scRNAseq capture. (**e**) Comparison of sex-biased genes from scRNAseq and snRNAseq reveals that several key genes exhibiting dichotomous expression across sexes as reported here are differentially captured by the two sequencing techniques. P values were calculated by Wilcoxon rank sum test. (**f**) There is a significant positive correlation between male defining genes in our scRNAseq and male-defining genes in Muto *et al.* (ref. 14) snRNAseq dataset (Spearman's rho = 0.7318, p val = 0.004129). scRNAseq = single-cell RNA sequencing, snRNAseq = single-nucleus RNA sequencing.

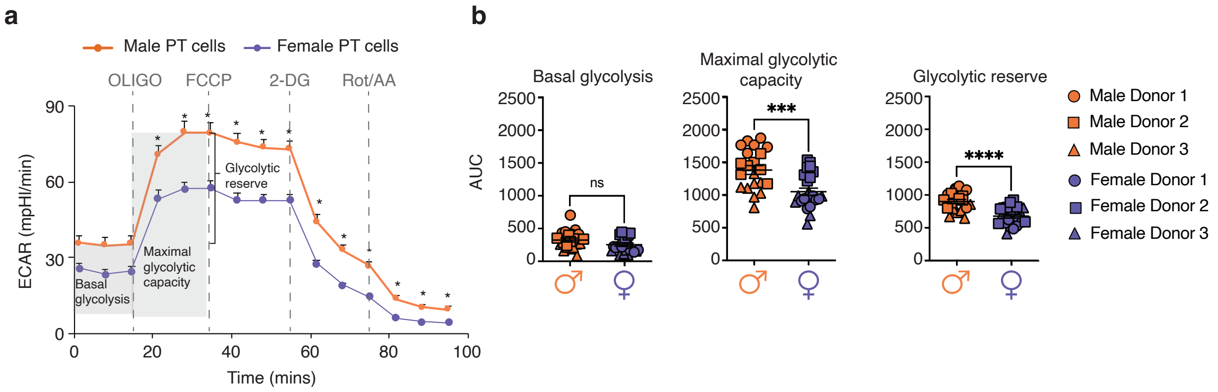

Supplementary Figure 7. Sex differences in the glycolytic rate of proximal tubular (PT) cells. (**a**) The extracellular acidification rate (ECAR) was monitored to assess the glycolytic metabolism of male (orange) and female (blue) PT cells at baseline and after metabolic stress. (n=3 donors/sex; n=21 replicates for males and 23 replicates for females). To induce metabolic stress, the following sequence of drugs was injected: 1µM oligomycin, 0.3µM FCCP, 100mM 2-DG, 1mM Rot/AA. Data are presented as mean values +/- SEM. (**b**) The basal glycolysis (p=0.063, u=162), maximal glycolytic capacity (p=0.0004, t=3.832, df=42), and glycolytic reserve (p<0.0001, t=5.331, df=42) of male (orange) and female (blue) PT cells were calculated from the ECAR curves in (**a**) (n=3 donors/sex; n=21 replicates for males and 23 replicates for females). Group-to-group differences were assessed using two-tailed unpaired t-test for variables following a normal distribution (maximal glycolytic capacity, glycolytic reserve), and Mann-Whitney tests for variables with a non-parametric distribution (basal glycolysis). Data are presented as mean values +/- SEM. *p<0.05; ***p<0.001; ****p<0.0001. PT, proximal tubule; AUC, area under the curve; ECAR, extracellular acidification rate; FCCP, p-trifluoromethoxy carbonyl cyanide phenyl hydrazone; 2-DG, 2-deoxyglucose; Rot, rotenone; AA: antimycin A.

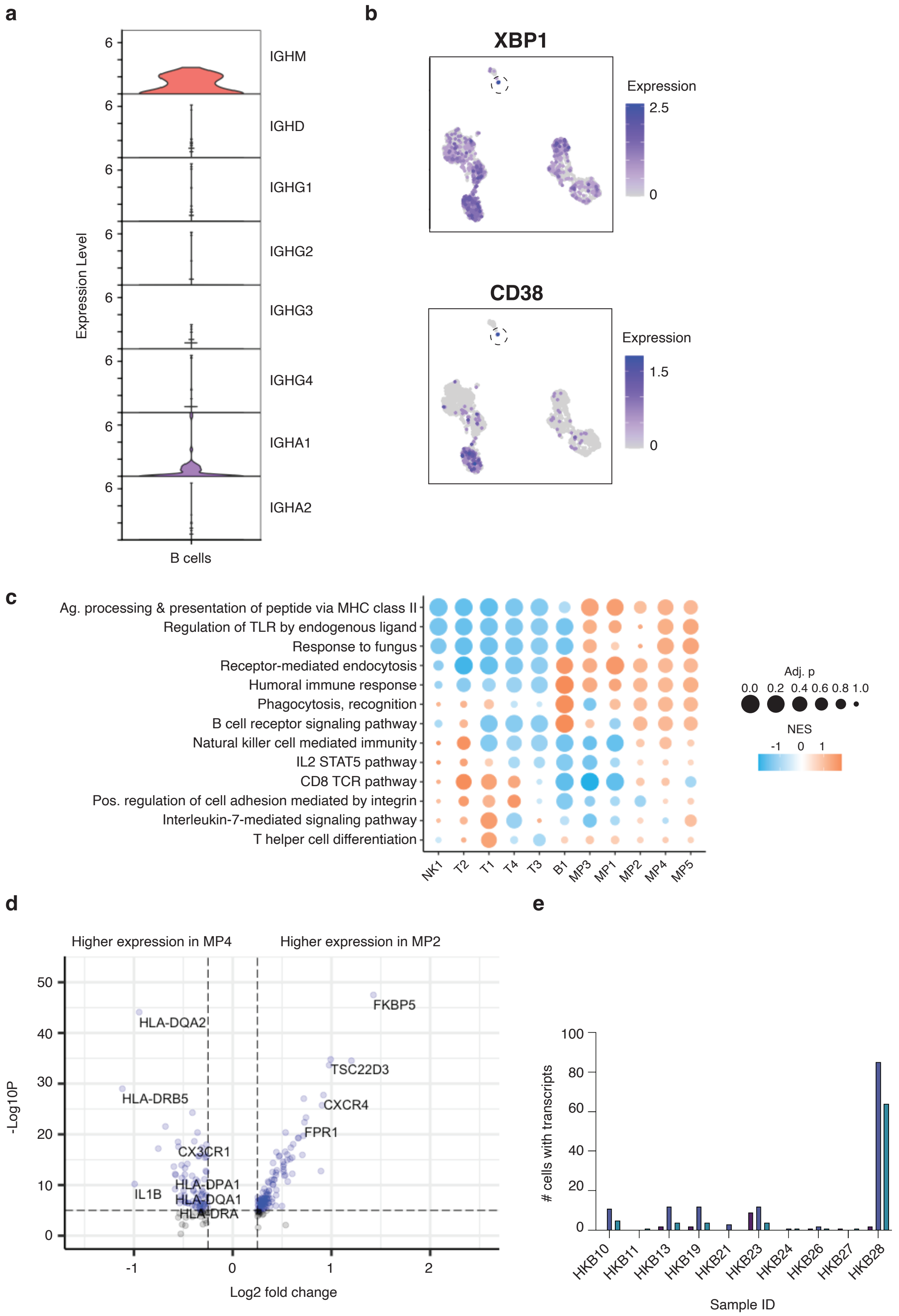

Supplementary Figure 8. Additional immune cell phenotyping data. (**a**)Expression of immunoglobulin heavy chain genes within the B cell cluster, showing low abundance of class-switched B cells in living donor kidney. No IGHE transcripts were detected. (**b**) Very few plasma cells marked by high *XBP1* and *CD38* expression were identified. (**c**) Pathway analysis summary for immune populations, indicating an enrichment in cell-type specific pathways in support of cluster annotations. P values were calculated by Wilcoxon rank sum with Bonferroni correction.(**d**) Differential gene expression between two clusters (MP2 and MP4) of $CD16^{+}$ monocyte-like cells identified an enrichment in antigen presentation genes in MP4, and differential expression of *CX3CR1* versus *CXCR4*. P values were calculated by Wilcoxon rank sum testing. (**e**) Expression of hemoglobin transcripts in the CD45-enriched sequencing datasets, prior to any quality control thresholds or data cleanup steps. Sample HKB28 had the highest abundance of cells positive for hemoglobin transcripts, suggesting more circulating cells in this sample.

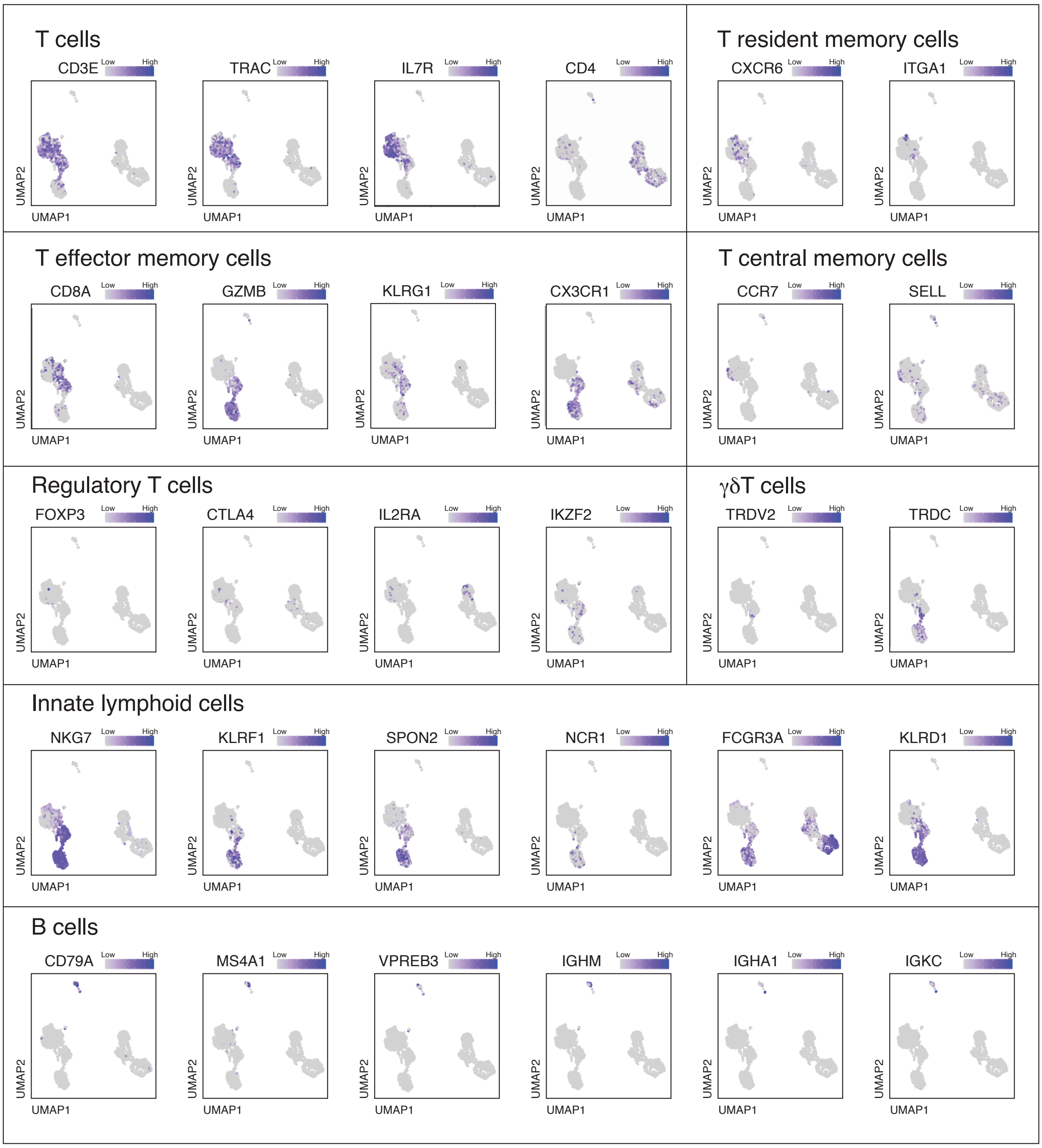

Supplementary Figure 9. Annotation of lymphocyte populations. Additional feature plots used to annotate of lymphocyte cell types including general T cell markers and subset-specific markers of T resident memory, T effector memory, and T central memory cells, as well as markers of regulatory T cells, γδT cells, innate lymphoid cells and B cells.

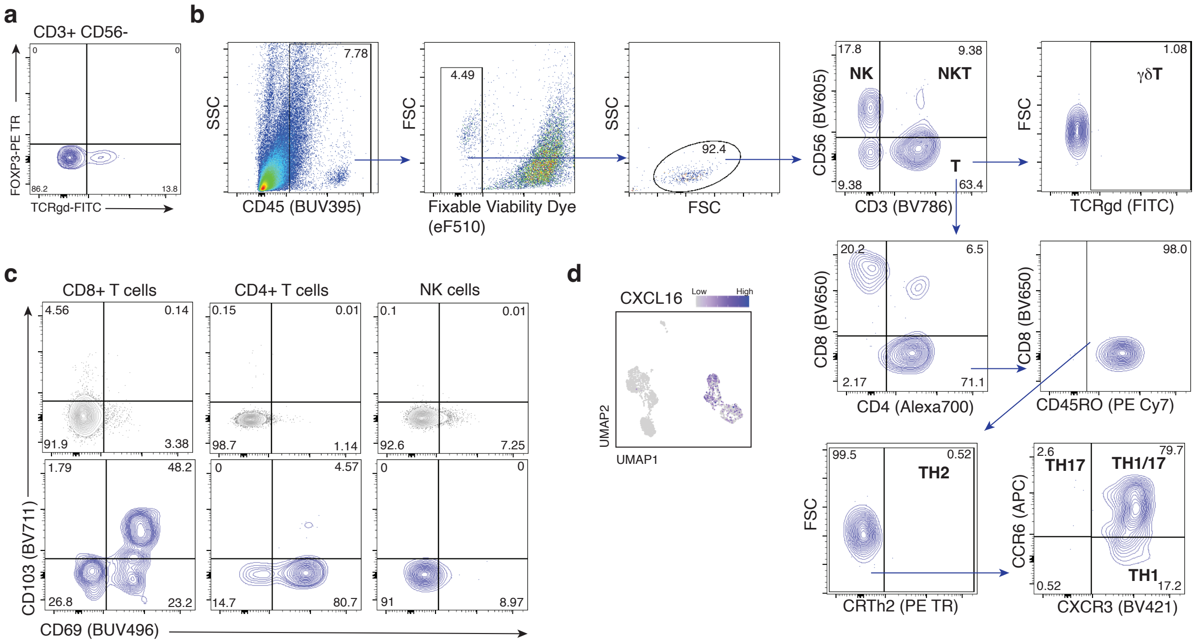

Supplementary Figure 10. Additional supporting data for the identification of resident signatures in kidney lymphocytes. (**a**) No FOXP3 expression was noted on T cells, and TCRγδ staining validated the presence of γδT cells within healthy kidney. (**b**) Gating strategy for the identification of T helper subsets. (**c**) Co-expression of CD69 and CD103, characteristic of Trm cells on $CD8^+$ and $CD4^+$ T cells and NK cells of the blood (grey, top row) versus kidney (blue, bottom row). (**d**) Expression of the chemokine CXCL16 in myeloid cells of the kidney supporting recruitment of $CXCR6^+$ lymphocytes. NK = Natural Killer cell, NKT = Natural Killer T cell, TH = T helper, FSC = Forward scatter, SSC = Side scatter.

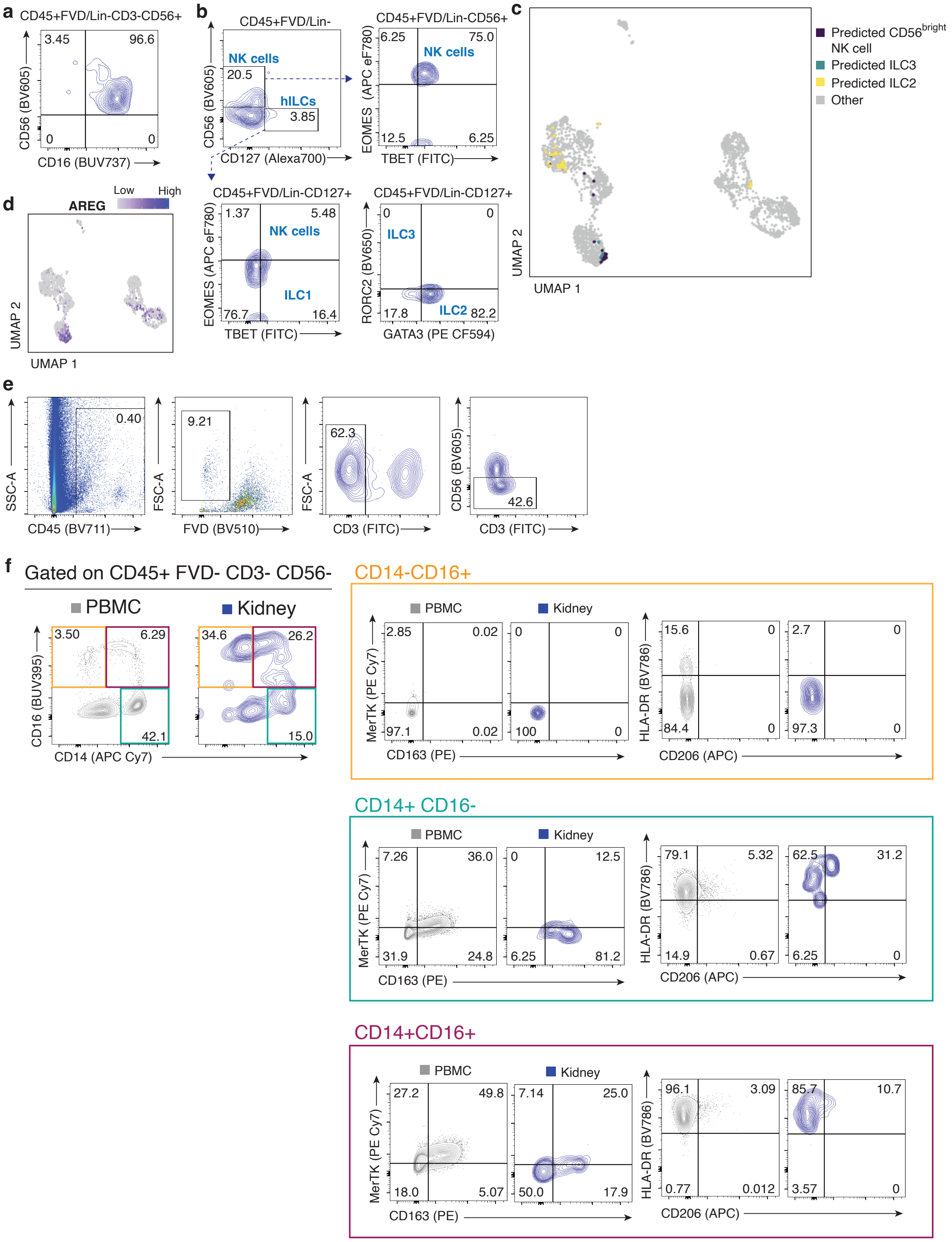

Supplementary Figure 11. Identification of innate lymphoid cell and myeloid populations in healthy human kidney. (**a**) The majority of NK cells within kidney are $CD56^{dim}CD16^{+}$, while (**b**) helper ILCs are present in very low abundance in kidney tissue. (**c**) Predictive identification of $CD56^{bright}CD16^{-}$ NK cells, ILC3s, and ILC2s within kidney immune transcriptomic data. (**d**) High expression of *AREG* encoding amphiregulin in kidney NK cells. (**e**) Gating strategy to remove lymphocytes from the population of interest. (**f**) Relative to blood, kidney tissue is enriched in $CD16^{+}$ myeloid populations, and also allowed for identification of a $CD14^{+}$ $CD206^{+}HLA\text{-}DR^{+}$ population likely representing MP1. FSC = Forward scatter, SSC = Side scatter, FVD = Fixable viability dye, NK = Natural Killer cell, ILC = Innate lymphoid cell, PBMC = Peripheral blood mononuclear cell.

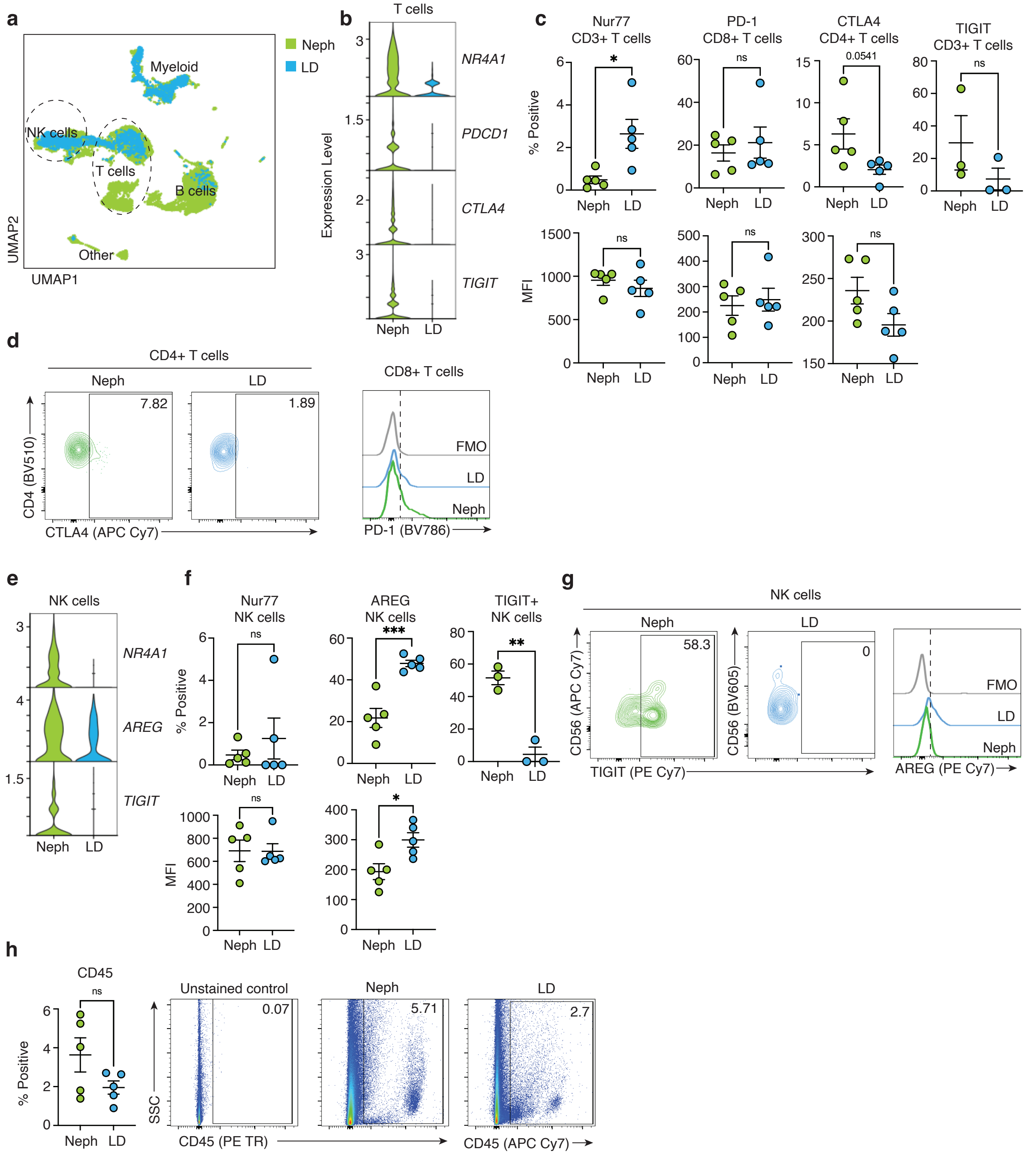

Supplementary Figure 12. Comparison of sequencing data from tumour nephrectomy versus living donor kidney specimens. (**a**) Integrated UMAP of kidney immune cells highlighting the contributions of cells derived from living donor (blue) versus unaffected kidney tissue from tumour nephrectomy (green). (**b**) Within the T cell compartment, the activation marker *NR4A1 (*encoding Nur77*)* along with checkpoint molecules *PDCD1 (*encoding PD-1*)*, *CTLA4* and *TIGIT* were more highly expressed in tumour nephrectomy data (green) than living donor (blue). (**c**) NR4A1 percent positivity (p=0.0152, t=3.076, df=8) and MFI (p=0.4206, u=8) by flow cytometry on $CD3^+$ T cells, PD-1 percent positivity (p=0.6905, u=10) and MFI (p=0.7024 t=0.3961 df=8) on $CD8^+$ T cells, CTLA-4 percent positivity (p=0.0541, t=2.256, df=8) and MFI (p=0.0851, t=1.964, df=8) on $CD4^+$ T cells and TIGIT percent positivity (p=0.2833, t=1.238, df=4) on $CD3^+$ T cells were compared between living donor (blue) (n=5) and tumour nephrectomy-derived (green) (n=5) T cells. Data are presented as mean values +/- SEM. (**d**) Representative plots of CTLA-4 on CD4+ T cells and PD-1 on CD8+ T cells of living donor (blue) and tumour nephrectomy-derived (green) cells. (**e**) NK cells exhibited similar trends at the transcript level with higher *NR4A1*, *AREG*, and *TIGIT* gene expression in tumour nephrectomy data (green) relative to living donor (blue). (**f**) While Nur77 protein was not differentially detected by percent positivity (p=0.5397, u=9) (n=5) or MFI (p>0.999, u=12) (n=5), AREG was higher in living donor (blue) NK cells by percentage (p=0.0006, t=5.420,

df=8) (n=5) and MFI (p=0.0182, t=2.959, df=8) (n=5), and TIGIT (p=0.0015, t=7.728, df=4) (n=3) was more highly detected on nephrectomy (green) NK cells. (Data are presented as mean values +/- SEM). (**g**) Representative plots of TIGIT and AREG expression differences in living donor and tumour nephrectomy tissue. (**h**) CD45+ cell elevation in tumour nephrectomy samples (green) did not reach significance (p=0.1129, t=1.780, df=8) (n=5), however, increased immune cell (CD45^{+}) abundance was observed in 3/5 tumour nephrectomy samples tested, with high donor heterogeneity in immune cell abundance was observed, indicative of greater differences in tissue microenvironment between tumour nephrectomy specimens relative to living donor (blue). Data are presented as mean values +/- SEM. Group-to-group differences were assessed using two-tailed unpaired t-test for variables following a normal distribution (NR4A1 % positive, PD-1 MFI, CTLA-4 % positive and MFI, TIGIT % positive, CD45 % positive) , and Mann-Whitney tests for variables with a non-parametric distribution (NR4A1 MFI, PD-1 % positive, Nur77 % positive and MFI). *p<0.05; **p<0.01;***p<0.001; ****p<0.0001. Neph = Unaffected kidney tissue from tumour nephrectomy, LD = Living donor, MFI = Median Fluoresence Intensity.

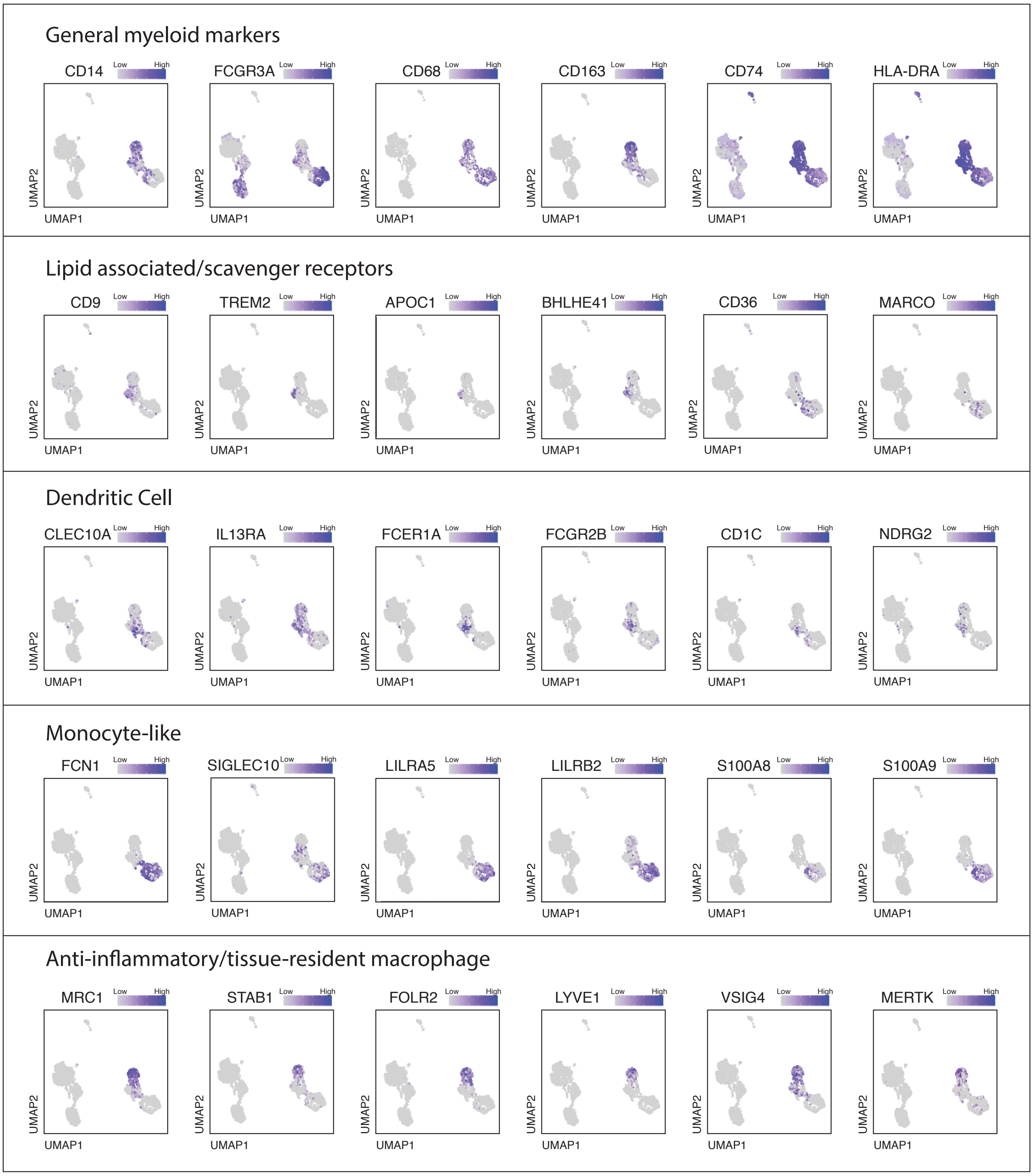

Supplementary Figure 13. Annotation of myeloid populations. Additional feature plots of myeloid cells supporting cell type annotations, highlighting general myeloid lineage markers, expression of scavenger receptors, and markers of dendritic cells, monocytes and macrophages.

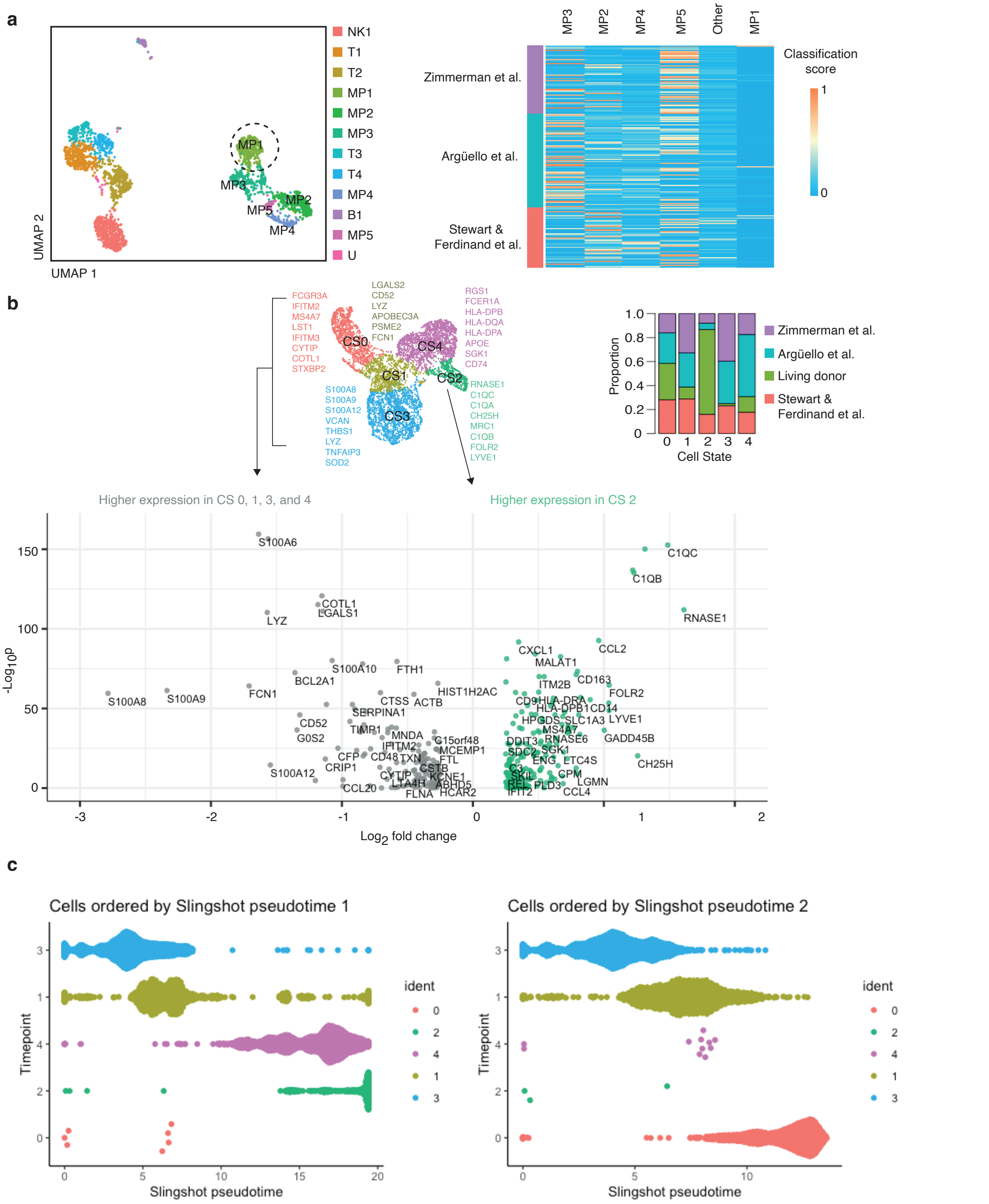

Supplementary Figure 14. Additional supporting plots for macrophage cell state identification. (**a**) Based on our identification of 5 clusters of myeloid lineage cells in living donor kidney, we used SingleCellNet to classify cells from previously published datasets[3,45,46] into our 5 cluster framework. Most cells captured in prior studies were classified as MP5 ($CD14^+$ monocytes), the smallest cluster in living donors; while MP1 (circled) the largest cluster in living donor data was scarcely represented in previously published data. (**b**) Merging the three datasets specified in (**a**) with our living donor dataset confirmed 5 cell states (CS) where living donor data comprised the majority of CS2. A volcano plot depicts genes enriched in CS2 versus the remaining four cell states, supporting that CS2 represents a resident alternatively-activated tissue macrophage population that is uniquely enriched in living donor kidney tissue. P values were calculated by Wilcoxon rank sum testing. (**c**)Slingshot pseudotime analysis supporting the annotation of CS1 as a transitional myeloid population across two suggested trajectories which placed CS2 and CS0 as the potential trajectory endpoints.

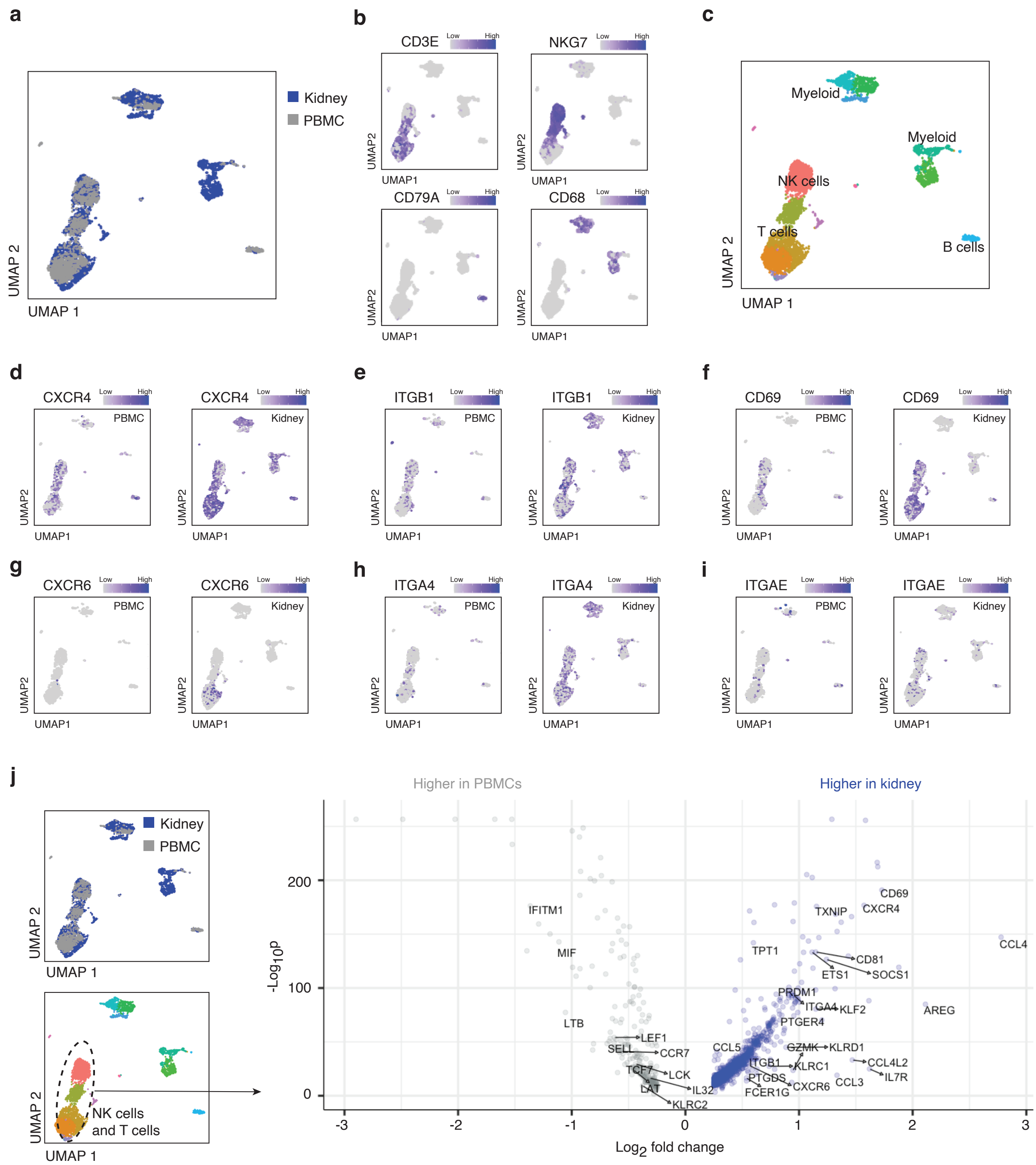

Supplementary Figure 15. Integration of PBMCs and kidney immune single-cell data. (**a**) scRNAseq data from living donor kidney immune cells and PBMCs[48] were integrated using Harmony. (**b**) Feature plots demonstrating expression of *CD3E*, *NKG7*, *CD79A*, and *CD68* used to annotate major immune populations in the combined dataset. (**c**) Annotation of major immune populations including T cells, NK cells, B cells and myeloid cells in the integrated PBMC and kidney immune dataset. Feature plots showing gene expression in PBMCs versus living donor kidney data of marker genes used for validation at the protein level including (**d**) *CXCR4*, (**e**) *ITGB1*, (**f**) *CD69*, (**g**) *CXCR6*, (**h**) *ITGA4* and (**i**) *ITGAE*. (**j**) Differential expression analysis of the T cells and NK cell clusters identifies genes which are upregulated in kidney lymphocytes and may represent kidney-adapted gene expression of these cells. P values were calculated by Wilcoxon rank sum testing.

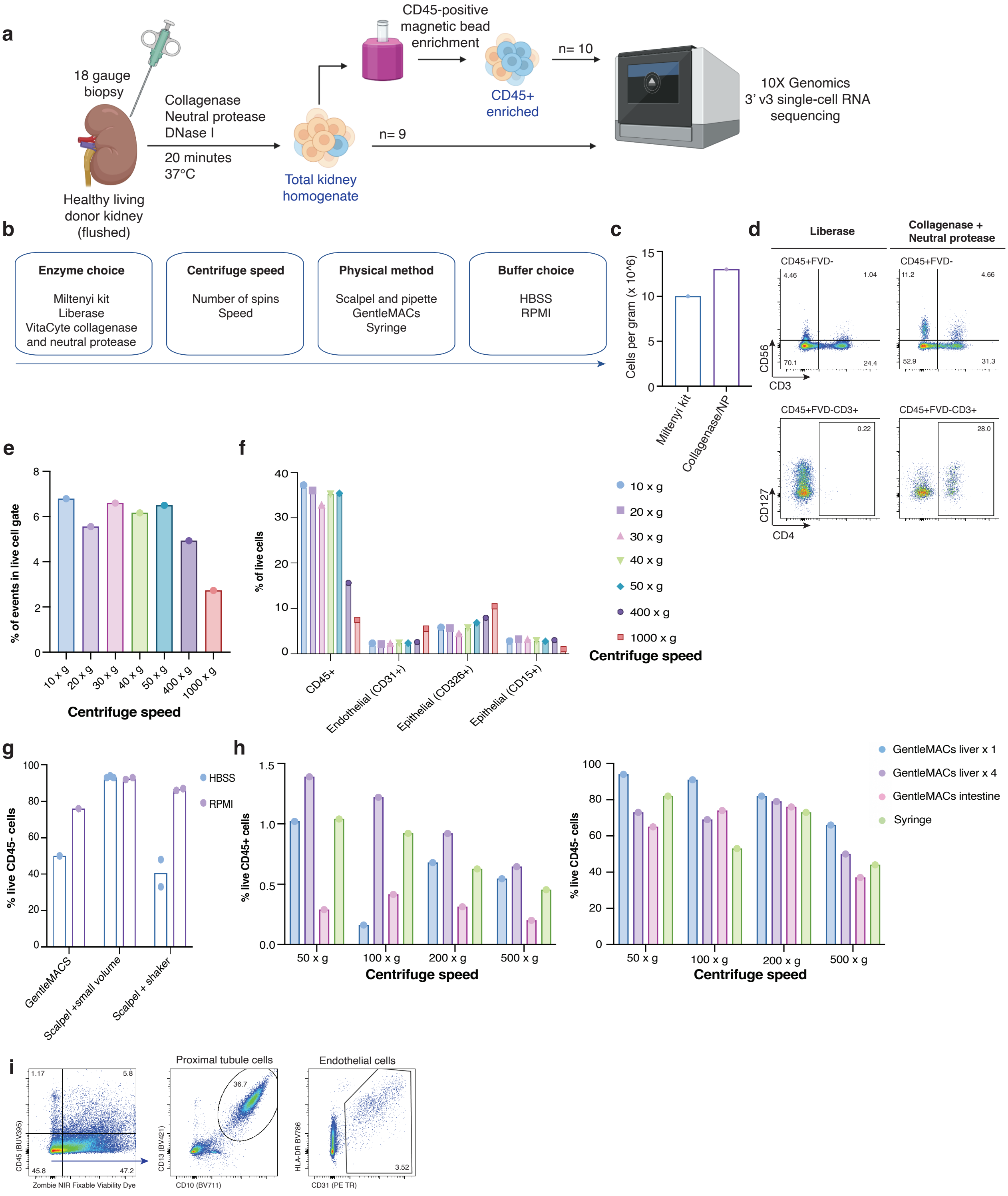

Supplementary Figure 16. Optimization of kidney tissue digestion protocol. (**a**) Final experimental protocol for generating single-cell RNA sequencing data from living donor kidney. (**b**) Workflow of options tested in determining the optimal digestion method. (**c**) Using mouse tissue, a commercial Miltenyi kidney digestion kit was compared to a collagenase and neutral protease mixture to compare yield and viability, with collagenase and neutral protease demonstrating superior yield and comparable viability. (**d**) Using unaffected human kidney from tumour nephrectomy tissue, Liberase was compared with collagenase and neutral protease, and flow cytometry was used to determine viability and cell phenotype, where it was determined that collagenase/neutral protease preserved key surface markers that appear to be cleaved by Liberase. (**e**) Fractions of dissociated human nephrectomy were centrifuged at different speeds to determine cell viability, which was reduced beyond speeds of 400 x g. (**f**) Using markers of key cell populations, by flow cytometry the contribution of different cell populations to each fraction by differential centrifugation determined that cell types were captured proportionally up to speeds of 400 x g. (**g**) To optimize yield and preservation of parenchymal cell viability, digestion in either HBSS or RPMI medium with collagenase and neutral protease was tested alongside physical methods of

dissociation including using GentleMACs, a scalpel and a small volume of dissociation medium (n=2), and a scalpel with incubation with constant agitation in a shaker (n=2). Over all methods, RPMI preserved parenchymal viability better than HBSS, while overall the greatest viability was in using a scalpel and small volume of dissociation medium. (**h**) Viability of immune ($CD45^+$) and parenchymal ($CD45^-$) populations across physical methods and centrifuge speeds to test whether the relative abundance of cell population viability changes with more aggressive physical dissociation, where generally more gentle dissociation preserved parenchymal cell viability whereas more aggressive physical dissociation improved yield of immune cells. Different Gentlemacs™Tissue dissociator settings named based on organ optimized for were tested (liver, intestine, etc). No clear change in fractionation was observed in differential centrifugation of the samples. n=1 unless otherwise specified. (**i**) Flow cytometric analysis of kidney parenchymal cells, depicting the capture of live proximal tubular epithelial cells ($CD10^+CD13^+$) and endothelial cells ($CD31^+HLA^-DR^{+/-}$).

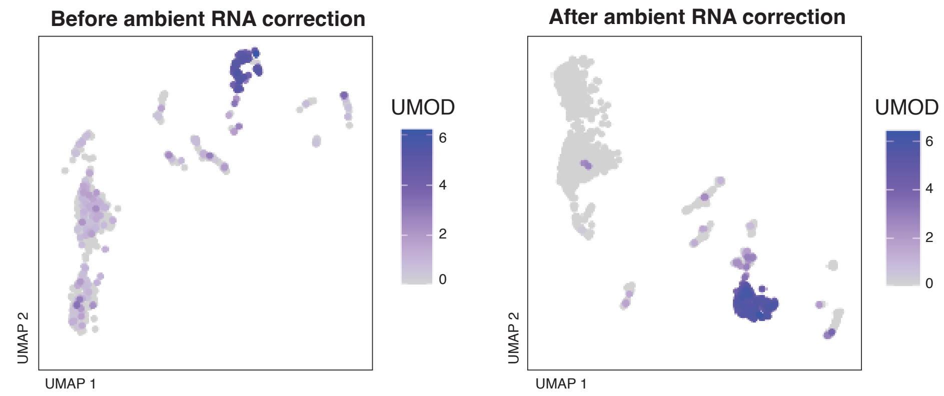

Supplementary Figure 17. Ambient RNA contamination. Feature plots showing the expression of UMOD, a gene specific to cTAL/LOH cells, with widespread low level expression present across all clusters prior to ambient RNA correction and more biologically appropriate expression patterns after ambient RNA correction, demonstrated with a sample dataset (Total9).

Supplementary Data

Supplementary Data 1. Results of sex analyses. Results of genes identified with Varimax rotated PCA, sPLS-DA, and differential gene expression analysis using MAST comparing male and female proximal tubular cells.

Supplementary Data 2. **DEGs snRNAseq vs. scRNAseq.** Differentially expressed genes (logFC > 0.25) in a sample processed by both single nucleus RNA sequencing and single cell RNA sequencing. Negative logFC values are more highly expressed in single cell RNA sequencing, while positive logFC values are more highly expressed in single nucleus RNA sequencing. P values were calculated by Wilcoxon rank sum testing with Bonferroni correction.

Supplementary Data 3. GSEA significant results. Summary of significant gene set enrichment analysis terms between male and female proximal tubular cells.

Supplementary Data 4. DEGs LD NK & T cells Vs PBMC. Results of differential gene expression analysis using Seurat comparing kidney NK and T lymphocytes to circulating lymphocytes (PBMCs) from two studies. P values were calculated by Wilcoxon rank sum testing with Bonferroni correction.

Supplementary Data 5. Cell-cell interactions Omnipath. Results of aggregate cell-cell communication inference with consensus and individual scores across methods, with Omnipath used as the reference interaction database.

Supplementary Data 6. Cell-cell interactions with complexes CellPhoneDB. Results of aggregate cell-cell communication inference with consensus and individual scores across methods, with CellPhoneDB used as the reference interaction database.

Supplementary Data 7. CellRanger summaries of sequenced samples. CellRanger summaries with sample metrics for each sequenced sample.

Supplementary Data 8. Curated cell annotation file. Curated marker gene list for cell type annotations.

Supplementary Data 9. Median gene expression by cluster. Summary table of median gene expression values for each cluster.

Supplementary Data 10. Average gene expression by cluster. Summary table of mean gene expression values for each cluster.

Supplementary Data 11. Median gene expression by cell type. Summary table of median gene expression values for each cell type.

Supplementary Data 12. Average gene expression by cell type. Summary table of mean gene expression values for each cell type.
