# Supplementary material for: Single-cell profiling of healthy human kidney reveals features of sex-based transcriptional programs and tissue-specific immunity
Source: Nat Commun. 2022 Dec 10;13:7634. doi: 10.1038/s41467-022-35297-z (PMC9741629; doi:10.1038/s41467-022-35297-z)
Supplement: Supplementary file 2 — Reporting Summary [file 41467_2022_35297_MOESM2_ESM.pdf]

nature portfolio

Corresponding author(s): Sarah Q. Crome
Ana Konvalinka

Last updated by author(s): Nov 10, 2022

Reporting Summary

Nature Portfolio wishes to improve the reproducibility of the work that we publish. This form provides structure for consistency and transparency in reporting. For further information on Nature Portfolio policies, see our Editorial Policies and the Editorial Policy Checklist.

Statistics

For all statistical analyses, confirm that the following items are present in the figure legend, table legend, main text, or Methods section.

n/a	Confirmed	
☐	☒	The exact sample size (*n*) for each experimental group/condition, given as a discrete number and unit of measurement
☐	☒	A statement on whether measurements were taken from distinct samples or whether the same sample was measured repeatedly
☐	☒	The statistical test(s) used AND whether they are one- or two-sided<br>*Only common tests should be described solely by name; describe more complex techniques in the Methods section.*
☒	☐	A description of all covariates tested
☐	☒	A description of any assumptions or corrections, such as tests of normality and adjustment for multiple comparisons
☐	☒	A full description of the statistical parameters including central tendency (e.g. means) or other basic estimates (e.g. regression coefficient) AND variation (e.g. standard deviation) or associated estimates of uncertainty (e.g. confidence intervals)
☐	☒	For null hypothesis testing, the test statistic (e.g. *F*, *t*, *r*) with confidence intervals, effect sizes, degrees of freedom and *P* value noted<br>*Give P values as exact values whenever suitable.*
☒	☐	For Bayesian analysis, information on the choice of priors and Markov chain Monte Carlo settings
☒	☐	For hierarchical and complex designs, identification of the appropriate level for tests and full reporting of outcomes
☒	☐	Estimates of effect sizes (e.g. Cohen's *d*, Pearson's *r*), indicating how they were calculated

Our web collection on statistics for biologists contains articles on many of the points above.

Software and code

Policy information about availability of computer code

Data collection

Single cells were isolated from peripheral blood or from pre-implantation biopsies from living donor kidneys after organ retrieval and flushing. Samples were washed twice in PBS plus 0.04% BSA, and viability was determined by a hemocytometer via trypan blue staining. Following counting, the appropriate volume for each sample was calculated for a target capture of 9000 cells. For CD45-enriched samples, all cells were sequenced. Cells were loaded onto a 10X single cell B chip. After droplet generation, samples were transferred onto a pre-chilled 96 well plate, heat sealed and incubated overnight in a Veriti 96-well thermocycler. The next day, sample cDNA was recovered using Recovery Agent provided by 10X and cleaned up using a Silane DynaBead mix as outlined by the user guide. Purified cDNA was amplified for 11 cycles before being cleaned up using SPRIselect beads. Samples were diluted 4:1 elution buffer:cDNA and run on a Bioanalyzer (Agilent Technologies) to determine cDNA concentration . cDNA libraries were prepared as outlined by the Single Cell 3' Reagent Kits v3 user guide with modifications to the PCR cycles based on the calculated cDNA concentration. The molarity of each library was calculated based on library size as measured by Bioanalyzer and qPCR amplification data (Roche). Samples were pooled and normalized to 1.5 nM. Library pool was denatured using 0.2N NaOH for 8 minutes at room temperature, and neutralized with 400 mM Tris-HCl. Library pools at a final concentration of 300pM were loaded to sequence on NovaSeq 6000 (Ilumina). Samples were sequenced with the following run parameters: Read 1-28 cycles, Read 2 - 90, Index1-10 cycles, Index 2- 10 cycles. Across samples, cells were sequenced to a target depth of 40,000 reads per cell. Mapping and quantification were performed using the 10X Genomics CellRanger pipeline version 3.1.0.

Flow cytometry data were collected using FACS Diva v8.0.1 on a BD LSR Fortessa flow cytometer.

Oxygen consumption rate and extracellular acidification rate were assessed for male and female proximal tubular epithelial cells using a SeaHorse XFe96 analyzer (Agilent).

Intracellular metabolite data was collected using liquid chromatography-mass spectrometry. RNA was quantified by Quant-iT Ribogreen assay

(Invitrogen). Pellets were dried in a turbovap and resuspended to 1 ug RNA to 0.5ul LC-MS grade water. Heavy-labelled (13C/15N) reference metabolites extracted from yeast were spiked in as an internal reference standard. Cellular metabolites were measured by injecting 2ul of sample in full scan MS1 mode using an Agilent 6550 qToF mass spectrometer coupled to an Agilent 1290 binary pump UPLC system. Metabolites were measured using an Agilent ZORBAX ExtendC18 1.8 um, 2.1 mm x 150 mm reverse phase chromatography column using tributylamine as an ion pairing agent. The Agilent 6550 qToF was fitted with a dual AJS ESI source and an iFunnel was a gas temperature set to 150C at 14L/min and 45 psig. Sheath gas temperature was set to 325C at 12L/min. Capillary and nozzle voltages were set to 2000V. Funnel conditions were changed from default to -30V DC, high pressure funnel drop -100V and RF voltage of 110V,low pressure funnel drop -50V and RF voltage of 60V. Metabolites were annotated by matching exact mass and retention time to an in-house database.

For gene expression validation, RNA was extracted from cell pellets using the RNAeasy Mini kit (Qiagen). RNA concentration was quantified in a Nanodrop instrument (Thermo) and 300-700ng of RNA were retrotranscribed to cDNA using the High-Capacity cDNA Reverse Transcription kit (Applied Biosystems). Gene levels were measured by real-time qPCR using a Power SYBR green PCR Master Mix reagent (Applied Biosystems) normalized to RPL31. The fluorescent signal was measured in a LightCycler 480 Instrument II (Roche).

Data analysis

Mapping and quantification were performed using the 10X Genomics CellRanger pipeline version 3.1.0 which excludes empty droplets, aligns reads, and generates feature-barcode matrices for each sample. The following software and algorithms were used as indicated in text and methods:

CellRanger version 3.1.0 10X Genomics https://support.10xgenomics.com/single-cell-gene-expression/software/downloads/latest
Rstudio Version 1.3.1093
Rstudio https://rstudio.com
biomaRt 2.42.1 Github https://github.com/grimbough/biomaRt
ChromXtractorPRO Rosebrock lab (adam.rosebrock@stonybrook.edu).
cowplot 1.1.1 CRAN https://github.com/wilkelab/cowplot
devtools 2.3.2 CRAN https://cran.r-project.org/web/packages/devtools/index.html
DoubletFinder 2.0.2 Github https://github.com/chris-mcginnis-ucsf/DoubletFinder
DropletUtils 1.6.1 Bioconductor https://bioconductor.org/packages/release/bioc/html/DropletUtils.html
Dplyr 1.0.4 CRAN https://cran.r-project.org/web/packages/dplyr/index.html
EnhancedVolcano 1.4.0 Bioconductor https://bioconductor.org/packages/release/bioc/vignettes/EnhancedVolcano/inst/doc/EnhancedVolcano.html
EnsDb.Hsapiens.v86 2.99.0 Bioconductor http://bioconductor.org/packages/release/data/annotation/html/EnsDb.Hsapiens.v86.html
ggbeeswarm 0.6.0 CRAN https://cran.r-project.org/web/packages/ggbeeswarm/index.html
ggplot2 3.3.3 CRAN https://cran.r-project.org/web/packages/ggplot2/index.html
ggthemes 4.2.4 CRAN https://cran.r-project.org/web/packages/ggthemes/index.html
harmony 1.0 Github https://github.com/immunogenomics/harmony
Hmisc 4.6-0 CRAN https://cran.r-project.org/web/packages/Hmisc/index.html
igraph 1.2.6 CRAN https://cran.r-project.org/web/packages/igraph/index.html
ktplots 1.1.7 Github https://github.com/zktuong/ktplots
liana 0.0.1 Github https://github.com/saezlab/liana
M3Drop 1.12.0 Github https://github.com/tallulandrews/M3Drop
MAST 3.12 Bioconductor https://www.bioconductor.org/packages/release/bioc/html/MAST.html
mixOmics 6.10.9 Bioconductor http://www.bioconductor.org/packages/release/bioc/html/mixOmics.html
OCAT Github https://github.com/bowang-lab/OCAT
presto 1.0.0 Github https://github.com/immunogenomics/presto
Patchwork 1.1.1 CRAN https://cran.r-project.org/web/packages/patchwork/index.html
RColorBrewer 1.1-2 CRAN https://cran.r-project.org/web/packages/RColorBrewer/index.html
reticulate 1.18-9006 CRAN https://cran.r-project.org/web/packages/reticulate/index.html
scater 1.14.6 Bioconductor https://bioconductor.org/packages/release/bioc/html/scater.html
scClustViz 1.3.8 Github https://github.com/BaderLab/scClustViz
scPred 1.9.0 Github https://github.com/powellgenomicslab/scPred
sctransform 0.3.2 CRAN https://cran.r-project.org/web/packages/sctransform/index.html
Seurat 3.2.3 CRAN https://cran.r-project.org/web/packages/Seurat/index.html
shiny 1.5.0 CRAN https://cran.r-project.org/web/packages/shiny/index.html
SingleCellExperiment 1.8.0 Bioconductor https://bioconductor.org/packages/release/bioc/html/SingleCellExperiment.html
SingleCellNet 0.1.0 Github https://github.com/pcahan1/singleCellNet
SingleR 1.10.0 Bioconductor https://bioconductor.org/packages/release/bioc/html/SingleR.html
slingshot 1.9.1 Github https://github.com/kstreet13/slingshot
SoupX 1.5.0 Github https://github.com/constantAmateur/SoupX
tidyverse 1.3.0 CRAN https://cran.r-project.org/web/packages/tidyverse/index.html
tsne 0.1-3 CRAN https://cran.r-project.org/web/packages/tsne/index.html
UCell 1.3.1 Bioconductor https://bioconductor.org/packages/release/bioc/html/UCell.html
umap 0.2.7.0 CRAN https://cran.r-project.org/web/packages/umap/index.html
uwot 0.1.10 CRAN https://cran.r-project.org/web/packages/uwot/index.html
VennDiagram 1.6.20 CRAN https://cran.r-project.org/web/packages/VennDiagram/index.html

ChEA3 https://maayanlab.cloud/chea3/

Scripts used for processing the raw data are deposited on Zenodo (DOI: 10.5281/zenodo.6633564).

For Flow Cytometry Analysis:
Flow cytometry data was recorded using BD FACSDiva software v8.0.1..
GraphPad Prism version 9.1.0 GraphPad software https://www.graphpad.com
FlowJo version 10.7.1 Tree Star https://www.flowjo.com

Schematics and kidney structure figure (Figure 1c) constructed using Biorender: https://biorender.com (licence to use provided)

Figures were assembled in Adobe Illustrator 2020: https://www.adobe.com/ca/products/illustrator.html

For manuscripts utilizing custom algorithms or software that are central to the research but not yet described in published literature, software must be made available to editors and reviewers. We strongly encourage code deposition in a community repository (e.g. GitHub). See the Nature Portfolio guidelines for submitting code & software for further information.

Data

Policy information about availability of data

All manuscripts must include a data availability statement. This statement should provide the following information, where applicable:

- Accession codes, unique identifiers, or web links for publicly available datasets
- A description of any restrictions on data availability
- For clinical datasets or third party data, please ensure that the statement adheres to our policy

The raw sequencing data generated in this study have been submitted to NCBI Gene Expression Omnibus under the accession number GSE202109, (https://www.ncbi.nlm.nih.gov/geo/query/acc.cgi?acc=GSE202109).

The final dataset (containing processed files) is also available online126 via: https://cells.ucsc.edu/?ds=living-donor-kidney.

Previously published sequencing data were accessed as follows: Stewart and Ferdinand et al.3 (https://data.humancellatlas.org/explore/projects/abe1a013-af7a-45ed-8c26-f3793c24a1f4); Muto et al.14, GSE151302 (https://www.ncbi.nlm.nih.gov/geo/query/acc.cgi?acc=GSE151302); Zimmerman et al.52, GSE128993 (https://www.ncbi.nlm.nih.gov/geo/query/acc.cgi?acc=GSE128993); Argüello et al.53, GSE159913 (https://www.ncbi.nlm.nih.gov/geo/query/acc.cgi?acc=GSE159913); Wang et al.55, GSE148665 (https://www.ncbi.nlm.nih.gov/geo/query/acc.cgi?acc=GSE148665); 10X Genomics Datasets (https://support.10xgenomics.com/single-cell-gene-expression/datasets/3.0.2/5k_pbmc_v3)56 and (https://support.10xgenomics.com/single-cell-gene-expression/datasets/2.1.0/t_4k)108; and Bernink et al.107, GSE114396 (https://www.ncbi.nlm.nih.gov/geo/query/acc.cgi?acc=GSE114396).

Additional public data repositories used for our analysis include CHEA3109 (https://maayanlab.cloud/chea3/) and the Bader lab repository of genesets: (http://download.baderlab.org/EM_Genesets/).

Human research participants

Policy information about studies involving human research participants and Sex and Gender in Research.

Reporting on sex and gender	Patient sex information was collected and considered in study design. Gender information was not collected. This is reported in Supplementary Table 2, as well as figures 2 and 3
Population characteristics	Refer to patient characteristics table - Supplemental Table 4. We designed the project to include female and male samples, and there was a range in age of donor samples from 33-65 years. No significant differences in average ages of male or female samples were observed.
Recruitment	Transplant patients receiving living donor kidneys were recruited and consented by SS, paying particular attention that the number of male and female donors was balanced. There was no obvious bias in the selection, and Toronto population is very ethnically diverse. However, UHN's patient pool is not necessarily representative of the broader human population and there may be additional cell types and states found from future studies from other centers. As part of eligibility for participation in living kidney donation, all donors of kidneys must be in good health. Expression profiles from captured cell types will vary to some extent with age, ethnicity, sex, and health status.
Ethics oversight	All experiments were conducted with institutional ethics approval from University Health Network (REB: 18-5914.0, Living donor; REB: 18-5489.0, Tumour nephrectomy), and informed consent was obtained from each recipient prior to surgery.

Note that full information on the approval of the study protocol must also be provided in the manuscript.

Field-specific reporting

Please select the one below that is the best fit for your research. If you are not sure, read the appropriate sections before making your selection.

☒ Life sciences ☐ Behavioural & social sciences ☐ Ecological, evolutionary & environmental sciences

For a reference copy of the document with all sections, see nature.com/documents/nr-reporting-summary-flat.pdf

Life sciences study design

All studies must disclose on these points even when the disclosure is negative.

Sample size	Sample size was not pre-determined, but equal proportions of males and females were recruited. Cell atlas completeness was assessed by the robust capture of expected cell types and the consistent capture of major cell types across samples, indicating that we captured

representative data with minimal heterogeneity between samples. Furthermore, based on the samples sizes from single cell RNA sequencing studies that had been reported prior to our study, 20 samples from 20 participants was a high number of samples comparitively

Data exclusions

Ambient RNA contamination was evident in the dataset (Supplementary Data Figure 2). To remove ambient RNA contamination, SoupX was used to estimate and remove contaminating counts using the AutoEst function.
DoubletFinder was used to identify cells most likely to be doublets and remove them from the dataset, rather than using a maximum gene or feature threshold. For CD45-enriched samples, fewer cells were collected overall compared to total kidney samples. For total samples, a high doublet rate threshold of 7.5% was applied (as described in the vignette and utilized in comparable studies), while for CD45-enriched samples, the doublet rate was calculated as 0.8% per 1000 cells captured, as per 10X Genomics estimated doublet rates (https://assets.ctfassets.net/an68im79xiti/4tjk4KvXzTWgTs8f3tvUjq/2259891d68c53693e753e1b45e42de2d/CG000183_ChromiumSingleCell3__v3_UG_Rev_C.pdf)
Cell type-specific thresholds were set to remove low quality cells from the data. For immune cell clusters (clusters expressing PTPRC), all cells with greater than 10% of UMIs mapped to mitochondrial genes were removed, along with cells that had low transcript abundance (less than 1000) or gene diversity (less than 200 unique genes). For parenchymal cells, all cells with greater than 40% of UMIs mapped to mitochondrial genes were removed, along with cells with low transcript abundance (less than 1000) and low relative gene diversity (less than 750 unique genes).
In addition to imposing cell-specific quality control thresholds, cells with transcripts corresponding to hemoglobin genes (HBB, HBA1 and HBA2) were removed.
1 male sample was excluded as data indicated possible sample contamination.

Replication

All flow and cell culture data was repeated in at least 3 independent individuals and, with number of n's indicated in figures. For scRNAseq studies, alll attempts at replication of were successful. Machine learning approaches to identify sex differences, as well as comparison of myeloid cell states made use of published independent data sets. We did note differences in populations observed in kidney compared to those in blood and reported these. Comparison of kidney lymphocytes to published PBMCs datasets was performed and replicated in 2 independent PBMC data sets. Additionally, we noted differences between immune populations in our dataset and other kidney datasets that used different tissue sources, and reported these findings in the manuscript. For machine learning approaches, we used 3 independent methods to identify conserved gene expression across independent methodologies, and all replication attempts were successful. Moreover in our kidney map, most clusters had representation from all sequenced samples, indicating findings were replicated across distinct study participants.

Randomization

Samples were not randomized, as each sample had to be analyzed fresh and immediately. We have controlled for the sex, but the overall sample size did not allow us to control for other potential confounders.

Blinding

Patients were de-identified and assigned an identifier. Researchers were aware of basic demographic information and clinicians were aware of clinical history.

Reporting for specific materials, systems and methods

We require information from authors about some types of materials, experimental systems and methods used in many studies. Here, indicate whether each material, system or method listed is relevant to your study. If you are not sure if a list item applies to your research, read the appropriate section before selecting a response.

Materials & experimental systems

n/a	Involved in the study	
☐	☒	Antibodies
☒	☐	Eukaryotic cell lines
☒	☐	Palaeontology and archaeology
☐	☒	Animals and other organisms
☒	☐	Clinical data
☒	☐	Dual use research of concern

Methods

n/a	Involved in the study	
☒	☐	ChIP-seq
☐	☒	Flow cytometry
☒	☐	MRI-based neuroimaging

Antibodies

Antibodies used

Cells were stained with the following surface antibodies: Anti-human CD8a FITC (1:100, clone RPA-T8, BioLegend, cat # 301050), Anti-human TCRgd FITC (1:100, clone B1, BioLegend, cat #331208), Anti-human CD3 FITC (1:100, clone UCHT1, BioLegend, cat # 300440), Anti-human CD8a PerCP (1:50, clone RPA-T8, BioLegend, cat # 301030), Anti-human CXCR6 PerCP Cy5.5 (1:50, clone K041E5, BioLegend, cat # 356010), Anti-human CCR8 PE (1:100, clone L263G8, BioLegend, cat #360604), Anti-human CD127 PE (1:50, clone hIL-7R-M21, BD Biosciences, cat # 557938), Anti-human CD15 PE (1:100, clone W6D3, BD Biosciences, cat # 562371), Anti-human CD163 PE (1:50,clone GHI/61, BioLegend, cat # 333606), Anti-human CD49d PE Dazzle 594 (1:100, clone 9F10, BioLegend, cat # 304325), Anti-human CRTh2 PE Dazzle 594 (1:50, clone BM16, BioLegend, cat #350126), Anti-human CD31 PE Dazzle 594 (1:100, clone WM59, BioLegend, cat # 303130), Anti-human CD16 PE Dazzle 594 (1:100, clone 3G8, BioLegend, cat # 302054), Anti-human CD45 PE-CF594 (1:100, clone HI30, BD Biosciences, cat # 562279), Anti-human CD29 PE Cy7 (1:100, clone TS2/16, BioLegend, cat # 303025), Anti-human CD45RO PE Cy7 (1:50, clone UCHL1, BD Biosciences,cat # 560608), Anti-human MerTK PE Cy7 (1:50, clone 590H11G1E3, BioLegend, cat # 367610), Anti-human TIGIT PE Cy 7 (1:50, clone MBSA43, Invitrogen, cat # 25-9500-42), Anti-human CD94 APC (1:100, clone HP-3D9, eBioscience, cat # 17-5094-42), Anti-human CCR6 APC (1:25, clone G034E3, BioLegend, cat # 353416), Anti-human CD206 APC (1:50, clone 15-2, BioLegend, cat # 321110), Anti-human CD4 Alexa700 (1:50, clone RPA-T4, eBioscience, cat # 56-0049-42), Anti-human CD127Alexa700 (1:50, clone eBioRDR5, eBioscience, cat #56-1278-42), Anti-human CXCR4 APC Cy7 (1:50, clone 12G5, BioLegend, cat # 306528), Anti-human CTLA-4 APC Cy7 (1:25, clone

BNI3, BioLegend,cat # 369634), Anti-human CD56 APC Cy7 (1:50, clone HCD56, BioLegend, cat # 318332), Anti-human CD45 APC Cy7 (1:100, clone HI30, BioLegend, cat # 304014), Anti-human CD14 APC eF780(1:100, clone 61D3, eBioscience, cat # 47-0149-42), Anti-human CXCR3 BV421 (1:50, clone G025H7, BioLegend, cat # 353716), Anti-human CD13 BV421 (1:50, clone WM15, BioLegend, cat #301716), Anti-human TCRgd BV510 (1:100, clone B1, BioLegend, cat # 331220), Anti-human TCRab BV510(1:100, clone IP26, BioLegend, cat # 306734), Anti-human CD5 BV510 (1:100, clone L17F12, BioLegend, cat # 364018), Anti-human FcER1 BV510 (1:100, clone AER-37, BioLegend, cat #334626), Anti-human CD303 BV510 (1:100, clone 201A, BioLegend, cat #354232), Anti-human CD123 BV510 (1:100, clone 6H6, BioLegend, cat # 306022), Anti-human CD34 BV510 (1:100, clone 581, BioLegend, cat #343528), Anti-human CD20 BV510 (1:100, clone 2H7, BioLegend, cat #302340), Anti-human CD3 BV510 (1:100, clone OKT3, BioLegend, cat # 317332), Anti-human CD14 BV510 (1:100, clone M5E2, BioLegend, cat # 301842), Anti-human CD19 BV510 (1:100, clone HIB19, BioLegend, cat # 302242), Anti-human CD4 BV510 (1:100, clone RPA-T4, BioLegend, cat # 300546), Anti-human CD56 BV605 (1:50, clone HCD56, BioLegend, cat # 318334), Anti-human CD69 BV650 (1:100, clone FN50, BioLegend, cat # 310934), Anti-human CD8a BV650 (1:50, clone RPA-T8, BioLegend, cat # 301042), Anti-human CD326 BV650 (1:100, clone 9C4, BioLegend, cat # 324226), Anti-human CD107a BV750 (1:50, clone H4A3, BioLegend, cat # 328638), Anti-human CD103 BV711 (1:100, clone Ber-ACT8, BioLegend, cat # 350222), Anti-human CD10 BV711 (1:100, clone HI10a, BioLegend, cat # 312226), Anti-human CD45 BV711 (1:100, clone HI30, BioLegend, cat # 304050), Anti-human CD3 BV785 (1:100, clone OKT3, BioLegend, cat # 317330), Anti-human HLA-DR BV785 (1:50, clone L243, BioLegend, cat # 307642), Anti-human PD-1 BV785 (1:50, clone EH12.2H7, BioLegend, cat # 329930), Anti-human CD45 BUV395 (1:100, clone HI30, BD Biosciences, cat #563792), Anti-human CD16 BUV395 (1:100, clone 3G8, BD Biosciences, cat # 563785), Anti-human CD3 BUV395 (1:100, clone UCHT1, BD Biosciences, cat # 563546), Anti-human CD69 BUV496 (1:50, clone FN50, BD Biosciences, cat # 750214), Anti-human CD16 BUV737 (1:100, clone 3G8, BD Biosciences, cat # 564434).
The following antibodies were used for intracellular staining: Anti-human TBET FITC (1:50, clone 4B10, BioLegend, cat # 644812), Anti-human Granzyme B FITC (1:100, clone QA16A02, BioLegend, cat # 372206), Anti-human Granzyme K PE (1:25, clone GM26E7, BioLegend, cat # 370512), Anti-human FOXP3 PE CF594 (1:25, clone 236A/E7, BD Biosciences, cat #563955), Anti-human GATA3 PE CF594 (1:25, clone L50-823, BD Bioscience, cat # 563510), Anti-human Amphiregulin PE Cy 7 (1:25, clone AREG559, Invitrogen, cat # 25-5370-42), Anti-mouse Nur77 APC (1:25, clone REA704, Miltenyi, cat # 130-111-231), Anti-human EOMES APC eF780 (1:25, clone WD1928, eBioscience, cat #47-4877-42), Anti-human RORgT BV650 (1:50, clone Q21-559, BD Biosciences, cat # 563424), Anti-human Perforin eF450 (1:100, clone dG9, Invitrogen, cat # 48-9994-42)

Validation

All antibodies for flow cytometry were validated against isotype controls in human cells by manufacturer for that application, see manufacturers' websites (noted above) for details. We further performed antibody titrations on human PBMCs or nephrectomy samples using fluorescence-minus-one controls, to account for autofluorescence of different immune and parenchymal populations, as well as identify the optimal dilution within our flow cytometry panel.

Animals and other research organisms

Policy information about studies involving animals; ARRIVE guidelines recommended for reporting animal research, and Sex and Gender in Research

Laboratory animals

All animals were species Mus musculus. Kidneys from female 8 week old C57BL/6 mice were used for initial digestion optimization experiments. Mice were ordered directly from Jackson Laboratories under Animal Use Protocol 6156 approved by the Toronto General Hospital Research Institute Animal Care Committee.

Wild animals

Study did not involve wild animals.

Reporting on sex

Sex was not considered in animal studies (used only for protocol optimization)

Field-collected samples

Study did not involve samples collected from the field.

Ethics oversight

All mouse experiments followed applicable regulations and guidelines and were approved by Toronto General Hospital Research Institutes Animal Care Committee (Animal Use Protocol 6156).

Note that full information on the approval of the study protocol must also be provided in the manuscript.

Flow Cytometry

Plots

Confirm that:

☒ The axis labels state the marker and fluorochrome used (e.g. CD4-FITC).

☒ The axis scales are clearly visible. Include numbers along axes only for bottom left plot of group (a 'group' is an analysis of identical markers).

☒ All plots are contour plots with outliers or pseudocolor plots.

☒ A numerical value for number of cells or percentage (with statistics) is provided.

Methodology

Sample preparation

Kidney biopsies were collected in RPMI 1640 (Gibco, cat # 11875119) on ice, and mechanically dissociated with a blade before enzymatic digestion at 37ºC with 0.1 mg/ml DNase I (STEMCELL, cat # 07470), 3300 CDA units/ml Collagenase MA (VitaCyte, cat # 001-2030) and 2990 NP units/ml BP neutral protease (VitaCyte, cat # 003-1000) for 20 minutes at 37ºC with intermittent agitation. Cell suspensions were filtered through 35µm cell strainer snap-cap FACS tubes (Falcon, cat# 352235) and a plunger from a 1ml syringe was used to gently mash remaining tissue in the strainer before rinsing strainer lid with 1:1

volume of FBS (HyClone, cat # SH3039603PM) on ice. After fresh tissue digestion, cells were washed in PBS + 2% FCS before staining. Cryopreserved cells were used for flow cytometry analysis of lymphocyte populations to control for any batch effects. Cells in these experiments were thawed and washed twice in PBS + 2% FCS.

Immune cells from peripheral blood for comparision were isolated using using a high density ficoll gradient. Briefly, peripheral blood was diluted 2-fold with PBS, carefully layered on an RT Ficoll gradient (Sigma HISTOPAQUE®-1119), and centrifuged at 400 x g for 30 minutes at room temperature. The buffy coat was carefully removed, diluted 5X-fold with PBS buffer, pelleted (300 x g, 5 minutes, 4°C), and resuspend in PBS + 2% FCS efore stainin. Clumps were separated by gentle pipetting to create a single cell suspension.

Cells were incubated at 4ºC for 15 minutes with an Fc receptor blocker (BioLegend TruStain FcX, cat # 422302) according to manufacturer instructions before cocktails of surface antibodies were added for 30 minutes at 4ºC. If intracellular targets/ transcription factors were included in the panel, cells were resuspended in FOXP3 transcription factor fix perm buffer (eBio, cat # 00-5523-00) and stained with intracellular antibodies in 1X permeabilization buffer (eBio, cat # 00-8333-56). If no intracellular targets were included in the staining panel, cells were fixed in 2% PFA (Thermo Scientific, cat # J19443) after surface staining.

Instrument

Experiments were performed on a BD LSR Fortessa flow cytometer

Software

Data were plotted using FlowJo v10.7.1 (TreeStar) and Prism (Graphpad, v9.1.0).

Cell population abundance

CD45+ (pan immune cell marker) in living donor kidney comprised 1.6% (range 0.89%-2.7%)of live cells by flow cytometry. Proportions of immune sub-populations detailed in Figures and Supplementary Figures, with source data provided as source data file.

Gating strategy

For immune cells, the order of gating was as follows: CD45+, Fixable Viability Dye-, FSC/SSC to exclude outliers. See Extended Data Figures 6b, 7b,e,f. Cell types were defined as follows: T cells (CD3+), NK cells (CD56+CD3-), CD8+ T cells (CD3+CD8+CD4-), CD4+ T cells (CD3+CD8-CD4+), Th1 cells (CD3+CD4+CD8-CD45RO+CXCR3+CCR6-), Th2 cells (CD3+CD4+CD8-CD45RO+CRTh2+), Th17 cells (CD3+CD4+CD8-CD45RO+CXCR3-CCR6+), Th1/17 cells (CD3+CD4+CD45RO+CXCR3+CCR6+), ILC1s (CD5-FCER1-CD303-CD123-CD34-CD20-TCRab-TCRgd-CD3-CD14-CD19-CD127+EOMES-TBET+), ILC2s (CD5-FCER1-CD303-CD123-CD34-CD20-TCRab-TCRgd-CD3-CD14-CD19-CD127+RORC2-GATA3+), ILC3s (CD5-FCER1-CD303-CD123-CD34-CD20-TCRab-TCRgd-CD3-CD14-CD19-CD127+ RORC2+GATA3-), Myeloid cells (CD3-CD56-CD14+/-CD16+/-).

☒ Tick this box to confirm that a figure exemplifying the gating strategy is provided in the Supplementary Information.
