# Supplementary material for: Single-cell profiling of healthy human kidney reveals features of sex-based transcriptional programs and tissue-specific immunity
Source: Nat Commun. 2022 Dec 10;13:7634. doi: 10.1038/s41467-022-35297-z (PMC9741629; doi:10.1038/s41467-022-35297-z)
Supplement: Supplementary file 3 — Description of Additional Supplementary Files [file 41467_2022_35297_MOESM3_ESM.pdf]

Description of Additional Supplementary Files

File Name: Supplementary Data 1
Description: Results of sex analyses.

File Name: Supplementary Data 2
Description: DEGs snRNAseq vs. scRNAseq.

File Name: Supplementary Data 3
Description: GSEA significant results.

File Name: Supplementary Data 4
Description: DEGs LD NK & T cells Vs PBMC.

File Name: Supplementary Data 5
Description: Cell-cell interactions Omnipath.

File Name: Supplementary Data 6
Description: Cell-cell interactions with complexes CellPhoneDB.

File Name: Supplementary Data 7
Description: CellRanger summaries of sequenced samples.

File Name: Supplementary Data 8
Description: Curated cell annotation file.

File Name: Supplementary Data 9
Description: Median gene expression by cluster.

File Name: Supplementary Data 10
Description: Average gene expression by cluster.

File Name: Supplementary Data 11
Description: Median gene expression by cell type.

File Name: Supplementary Data 12
Description: Average gene expression by cell type.
